# DNA methylation by CcrM activates the transcription of two genes required for the division of *Caulobacter crescentus*

**DOI:** 10.1111/mmi.12180

**Published:** 2013-03-11

**Authors:** Diego Gonzalez, Justine Collier

**Affiliations:** Department of Fundamental Microbiology, Faculty of Biology and Medicine, University of LausanneQuartier UNIL/Sorge, Lausanne, CH 1015, Switzerland

## Abstract

DNA methylation regulates many processes, including gene expression, by superimposing secondary information on DNA sequences. The conserved CcrM enzyme, which methylates adenines in GANTC sequences, is essential to the viability of several *Alphaproteobacteria*. In this study, we find that *Caulobacter crescentus* cells lacking the CcrM enzyme accumulate low levels of the two conserved FtsZ and MipZ proteins, leading to a severe defect in cell division. This defect can be compensated by the expression of the *ftsZ* gene from an inducible promoter or by spontaneous suppressor mutations that promote FtsZ accumulation. We show that CcrM promotes the transcription of the *ftsZ* and *mipZ* genes and that the *ftsZ* and *mipZ* promoter regions contain a conserved CGACTC motif that is critical to their activities and to their regulation by CcrM. In addition, our results suggest that the *ftsZ* promoter has the lowest activity when the CGACTC motif is non-methylated, an intermediate activity when it is hemi-methylated and the highest activity when it is fully methylated. The regulation of *ftsZ* expression by DNA methylation may explain why CcrM is essential in a subset of *Alphaproteobacteria*.

## Introduction

DNA methylation regulates many processes in eukaryotes and prokaryotes by superimposing secondary information on the DNA sequence. N6-methyl adenines are found in the genomes of many eubacteria, archaebacteria, fungi and protists (Wion and Casadesus, 2006; Marinus and Casadesus, 2009). In eubacteria, most DNA adenine methyltransferases are part of restriction/modification systems, protecting bacterial genomes from a restriction enzyme companion. Solitary DNA adenine methyltransferases are also found in many bacterial species in the absence of a cognate endonuclease. The DNAmethyltransferase Dam of *Gammaproteobacteria* and the cell cycle-regulated DNA methyltransferase CcrM of *Alphaproteobacteria* are such examples, methylating adenines in GATC and GANTC sequences respectively (Zweiger *et al*., 1994; Stephens *et al*., 1996; Berdis *et al*., 1998; Wion and Casadesus, 2006; Albu *et al*., 2012). Dam is conserved in a subset of *Gammaproteobacteria* and CcrM in all sequenced *Alphaproteobacteria* except *Rickettsiales*.

In *Gammaproteobacteria*, the conservation of Dam may be explained by its involvement in several key cellular processes, such as DNA repair, transcriptional regulation or the initiation of DNA replication (Wion and Casadesus, 2006; Low and Casadesus, 2008; Collier, 2009; Marinus and Casadesus, 2009). All of these processes rely on the periodic variation in the methylation state of the adenines in GATC sequences that occurs upon DNA replication. Since Dam is present and active throughout the cell cycle in most, if not all, *Gammaproteobacteria*, newly incorporated adenines become methylated shortly after replication (Low and Casadesus, 2008; Collier, 2009). Certain GATC sites are located in promoter regions and are important components of epigenetic mechanisms regulating gene expression. Well-characterized examples of genes regulated by such epigenetic mechanisms include *agn43* and *sci1* and the *pap* and *gtr* operons in enterobacteria; all involve specific transcription factors (Lrp, OxyR and Fur), whose DNA binding activities affect and are affected by the methylation state of promoter regions (Wion and Casadesus, 2006; Low and Casadesus, 2008; Peterson and Reich, 2008; Broadbent *et al*., 2010; Kaminska and van der Woude, 2010; Brunet *et al*., 2011). In several *Gammaproteobacteria*, methylation by Dam is essential for cell viability. In *Vibrio cholera*, for example, Dam methylation is required for the replication of one of the chromosomes (Demarre and Chattoraj, 2010; Koch *et al*., 2010; Val *et al*., 2012). In other *Gammaproteobacteria*, methylation by Dam is often dispensable in non-stressed growth conditions.

CcrM was first described and has mainly been studied in *Caulobacter crescentus* (Zweiger *et al*., 1994; Stephens *et al*., 1996; Collier, 2009). *C. crescentus* divides asymmetrically, giving a motile swarmer cell and a sessile stalked cell (Curtis and Brun, 2010). A swarmer cell needs to start differentiating into a stalked cell before it can initiate the replication of its chromosome, which happens only once per cell cycle (Marczynski, 1999; Collier, 2012). Stalked cells immediately start the replication of their chromosomes. In *C. crescentus*, CcrM-directed methylation takes place only in late pre-divisional cells (Zweiger *et al*., 1994; Marczynski, 1999). Once the replication fork passes through a GANTC site, the two new copies of this site remain hemi-methylated until the end of chromosome replication in pre-divisional cells, when the newly synthesized strand gets methylated by CcrM giving fully methylated DNA (Zweiger *et al*., 1994; Marczynski, 1999). The duration of the period during which a locus stays hemi-methylated is dependent on its position on the chromosome: loci that are close to the origin remain hemi-methylated for the longest period of time during the cell cycle after their replication (Collier, 2009). These periodic switches from fully to hemi-methylated DNA during the cell cycle have been assimilated to a molecular clock, allowing the sequential activation or repression of some genes ordered along the chromosome from the origin to the terminus (Reisenauer *et al*., 1999; Reisenauer and Shapiro, 2002; Collier *et al*., 2007; Low and Casadesus, 2008; Collier, 2009).

CcrM methyltransferases were shown to be essential for the viability of each *Alphaproteobacterium* where this was tested (*C. crescentus*, *Rhizobium meliloti*, *Agrobacterium tumefaciens* and *Brucella abortus*), at least in the growth conditions that were chosen to make these experiments (Stephens *et al*., 1996; Wright *et al*., 1997; Robertson *et al*., 2000; Kahng and Shapiro, 2001). So far, the reasons why CcrM can be essential in *Alphaproteobacteria* are not understood. The periodic switches from fully to hemi-methylated DNA are not essential in *C. crescentus*, since a strain that maintains its chromosome fully methylated throughout the cell cycle is still viable (Zweiger *et al*., 1994; Wright *et al*., 1996; Collier *et al*., 2007). Similarly, *R. meliloti, A. tumefaciens* and *B. abortus* are still viable when CcrM is overproduced (Wright *et al*., 1997; Robertson *et al*., 2000; Kahng and Shapiro, 2001). In *C. crescentus*, the expression of two cell cycle-regulated genes, *dnaA* and *ctrA*, encoding two essential regulators of the *C. crescentus* cell cycle, seems to be modulated by the methylation of adenines in GANTC motifs present in their promoter regions (Reisenauer and Shapiro, 2002; Collier *et al*., 2007; Collier, 2009). Successful mutagenesis of these methylation sites on the chromosome nevertheless demonstrated that none of these are essential for the viability of *C. crescentus* (Reisenauer and Shapiro, 2002; Collier *et al*., 2007), suggesting that the methylation of the *dnaA* or the *ctrA* promoters is not the essential activity of CcrM. No methylation-dependent transcriptional regulator modulating the transcription of these two genes has been identified so far.

Before CcrM-depleted cells die in rich medium, they form long and smooth filaments, indicating that an early step during the cell division process is inhibited when the chromosome is not methylated by CcrM (Stephens *et al*., 1996). In most bacterial cells, a multiprotein complex called the divisome mediates cytokinesis (Adams and Errington, 2009). The essential FtsZ protein, a tubulin-like GTPase, polymerizes into a ring-like structure at the future division site (Bi and Lutkenhaus, 1991; Margolin, 2005; Harry *et al*., 2006). This Z-ring acts as a scaffold for the assembly of the rest of the divisome (Margolin, 2005; Adams and Errington, 2009; Goley *et al*., 2011) and provides the mechanical force for cell division (Li *et al*., 2007). In *C. crescentus*, the assembly of the Z-ring is spatially regulated by the MipZ protein, which co-ordinates the initiation of chromosome replication with cell division (Thanbichler and Shapiro, 2006; Kiekebusch *et al*., 2012). MipZ interacts with the partitioning protein ParB, which in turn binds to the *parS* locus near the chromosomal origin. When the replication of the chromosome initiates, one copy of the newly replicated origin is rapidly segregated to the opposite cell pole, while the other remains at the stalked pole of the cell (Jensen and Shapiro, 1999; Viollier *et al*., 2004). The bipolar subcellular localization of MipZ promotes Z-ring assembly near mid-cell, by directly inhibiting FtsZ polymerization near the cell poles (Thanbichler and Shapiro, 2006). *C. crescentus* cells depleted for FtsZ or MipZ form smooth filaments, demonstrating the early requirement for FtsZ and MipZ during the cell division process (Wang *et al*., 2001; Thanbichler and Shapiro, 2006).

In this work, we show that the transcription of the *ftsZ* and *mipZ* genes is strongly downregulated in cells that lack the CcrM DNA adenine methyltransferase and that FtsZ levels are limiting for cell division, solving the long-standing question on why CcrM is essential for cell division and for the viability of *C. crescentus* cells cultivated in rich medium. We also find that the *ftsZ* and *mipZ* promoter regions contain conserved CGACTC motifs that are critical to their activities and to their efficient activation by CcrM. We use a novel method to test if the *ftsZ* and *mipZ* promoters are more active when the conserved CGACTC motifs in these promoters are artificially hemi-methylated in Δ*ccrM* cells. Our results suggest that the methylation of the *ftsZ* and *mipZ* promoters stimulates their activity. The activation of *ftsZ* and *mipZ* transcription by CcrM may provide an explanation for the phylogenetic conservation of the *ccrM* gene in *C. crescentus* and in other related *Alphaproteobacteria*.

## Results

### *C. crescentus* cells lacking CcrM are elongated but nevertheless viable in minimal medium

Previous attempts to isolate a *C. crescentus* Δ*ccrM* strain on rich medium were unsuccessful, suggesting that the *ccrM* gene may be essential for the viability of *C. crescentus* (Stephens *et al*., 1996). Further analysis using a conditional *ccrM* mutant strain (LS2144), where the only copy of the *ccrM* gene is under the control of the xylose-inducible *xylX* promoter, also supported this conclusion: CcrM-depleted cells grown in rich medium (PYE) containing 0.2% glucose became very filamentous and viability counts decreased sharply within several hours (Stephens *et al*., 1996). To test the possibility that the essentiality of the *ccrM* gene may be dependent on growth conditions, we cultivated the LS2144 strain in minimal medium (M2G) lacking the xylose inducer. We observed that the LS2144 cells were only slightly elongated (Fig. S1), indicating that the cell division defect is attenuated in minimal medium, compared with rich medium. We confirmed that the same strain cultivated in rich medium containing 0.2% glucose and lacking the xylose inducer became filamentous and lost viability as previously described (Stephens *et al*., 1996) (data not shown). To clearly demonstrate that *ccrM* was not essential in minimal medium, we tried to construct a Δ*ccrM* mutant strain by transduction of the Δ*ccrM* mutation from the LS2144 strain into the wild-type strain using M2G as the selective medium. We found that transduction of the Δ*ccrM* mutation into the wild-type strain and into the wild-type strain containing pSC226 expressing *ccrM* from the *xylX* promoter, was comparable (Fig. S2). This observation suggested that the isolation of a Δ*ccrM* strain was not dependent on the appearance of a suppressor mutation. We also showed that the chromosome of the Δ*ccrM* strain (JC1149) that we isolated was efficiently digested by the HinfI restriction enzyme, demonstrating that GANTC sites were not methylated, as expected for Δ*ccrM* cells (Fig. S3). These results show that the *ccrM* gene is not essential for viability in minimal medium.

**Fig. 1:**
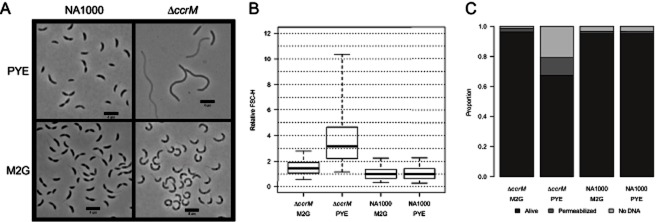
Cells lacking CcrM are elongated but nevertheless viable in minimal medium. NA1000 (wild-type) and JC1149 (Δ*ccrM*) cells were harvested from exponential liquid cultures in rich (PYE) or minimal (M2G) media. A. Morphologies of the cells visualized by phase-contrast microscopy. The black bars are scales indicating 4 μm on the images. B. Cell size distributions in NA1000 and Δ*ccrM* populations, as determined by flow cytometry. Forward scattering values (FSC-H) were used as an estimate of cell sizes. The median is shown as a bold line; the limits of the box are the first and the third quartile; the whiskers represent the 5% and 95% quantiles. A total of 20 000 cells were analysed for each population. C. Proportion of dead or damaged cells in NA1000 and Δ*ccrM* populations. Cells were stained with 5 μg ml^−1^ propidium iodide (PI) and 5 μg ml^−1^ 4′,6-diamidino-2-phenylindole (DAPI) and then visualized by fluorescence microscopy. Cells giving a signal for DAPI and not for PI were considered alive; cells giving a signal for PI were considered permeable; cells giving no signal with either DAPI or PI were considered as containing no DNA. More than 200 cells were analysed for each population.

The Δ*ccrM* strain reached high densities when cultivated in rich and minimal liquid media, although cells had very different morphologies in each condition. In minimal medium, most of the Δ*ccrM* cells were thinner and on average approximately 1.5-fold longer than wild-type cells (Fig. 1A and B). Less than 5% of the Δ*ccrM* cells were more than threefold longer than the median wild-type cell. Strikingly, the very low proportion of dead cells in the Δ*ccrM* population was comparable to that observed for the wild-type population grown in the same conditions (Fig. 1C and Fig. S4), confirming that the loss of CcrM does not affect viability in minimal medium. As expected, Δ*ccrM* cells had a much more severe phenotype when cultivated in rich medium at 28°C: most Δ*ccrM* cells were filamentous with high cell length variability within the population and with frequent membrane defects (Fig. 1). About 35% of the cells in the Δ*ccrM* population cultivated in rich medium were dead. These either contained no DNA, as visualized by DAPI staining, or were permeable to the dead cell stain propidium iodide (Fig. 1C and Fig. S4). Overall, the phenotypes of the Δ*ccrM* cells in rich medium at 28°C were consistent with severe inhibition of cell division associated with membrane integrity defects, sometimes leading to cell death. We also observed that Δ*ccrM* cells cultivated in rich medium at 22°C instead of 28° were very elongated but with viabilities comparable to that of the wild-type strain (data not shown). The phenotype of Δ*ccrM* cells cultivated in twofold or fourfold diluted rich medium also improved: the median cell length was shorter and membrane problems were less frequent than when cells were cultivated in non-diluted rich medium (data not shown). These observations suggest that the phenotype of Δ*ccrM* cells is more severe in conditions that promote fast growth.

### *C. crescentus* cells lacking CcrM contain low levels of the two cell division proteins FtsZ and MipZ

**Fig. 2:**
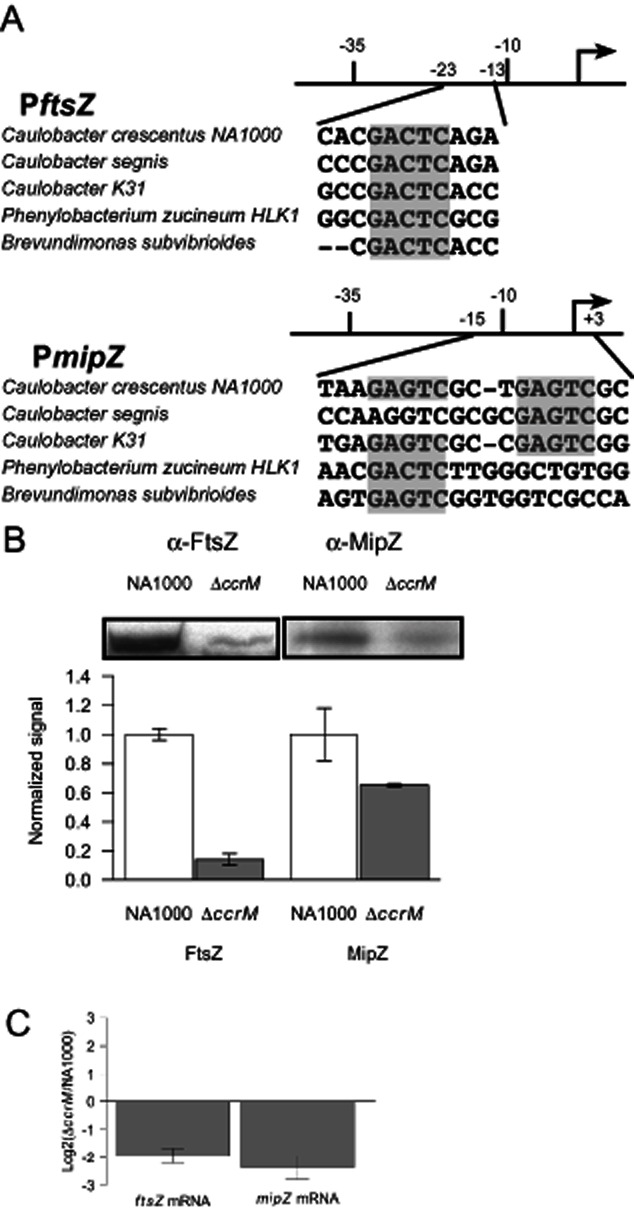
Cells lacking CcrM accumulate low levels of *ftsZ* and *mipZ* mRNAs, leading to low levels of FtsZ and MipZ proteins. A. Schematics, partial sequences and phylogenetic conservation of the *ftsZ* and *mipZ* promoter regions containing conserved GANTC motifs. The schematics show the position of the GANTC motifs (highlighted in grey) upstream of the +1 transcription start sites (Kelly *et al*., 1998; McGrath *et al*., 2007). The multiple alignments below compare the promoter sequences surrounding the conserved GANTC motifs in *Caulobacter crescentus*, and in its four closest sequenced relatives *Caulobacter segnis, Caulobacter K31, Phenylobacter zucineum* and *Brevundimonas subvibrioides*. B. Immunoblot analysis comparing the intracellular levels of FtsZ (Mohl *et al*., 2001) and MipZ (Thanbichler and Shapiro, 2006) in NA1000 and JC1149 (Δ*ccrM*) cells. Cells extracts were prepared from exponential phase cells cultivated in M2G medium. The graphs below the images show relative signal quantifications using images obtained using cell extracts from minimum two independent cultures; the normalization factor is the average of NA1000 signal quantification values for each protein. The OD_660_ was used to normalize the global protein content in each cell extract; to compensate for possible biases in OD_660_ values due to differences in cell shape and length, a stable non-specific protein signal also detected by immunoblot was used as a second normalization factor for the relative quantification of blots. C. Quantitative real-time PCR analysis comparing *fstZ* and *mipZ* mRNA levels in NA1000 cells compared with JC1149 cells. Cell extracts were prepared from cells cultivated in exponential phase in M2G medium. *ftsZ* and *mipZ* mRNA levels were quantified using NA1000 as the calibrator and the levels of the *CC_3527* mRNA as an internal reference. The graph shows the log_2_ values of the average ratios of *ftsZ* or *mipZ* mRNA levels in JC1149 and NA1000 cells. Error bars correspond to the standard deviations from three independent biological samples for each strain.

We hypothesized that DNA methylation could directly regulate the transcription of one or more genes involved in cell division. To identify potential regulatory targets of CcrM, we performed a bioinformatic search, looking for sets of orthologous genes encoding proteins known to be directly or indirectly involved in cell division and whose promoter region contained at least one GANTC site in *C. crescentus* and in six other closely related *Alphaproteobacteria*. We considered that the conservation of a GANTC site provided an indication that it might perform a regulatory function important enough to be conserved across species. We found that the *ftsZ*, *mipZ* and *ftsE* genes had such strictly conserved GANTC methylation sites (Table S1). In this study, we focused on the first two genes, because they are required earlier during the division process, being involved in the crucial cell division plane selection process (Wang *et al*., 2001; Thanbichler and Shapiro, 2006; Goley *et al*., 2011). In addition, the *ftsZ* and *mipZ* promoter regions share a conserved CGACTC motif (on the forward and the reverse strands, respectively, in *C. crescentus*) that is located at similar distances from their translation start sites in most examined bacterial species (Fig. 2A and Tables S2 and S3). We therefore hypothesized that these two genes may be co-regulated in *Alphaproteobacteria*, in a manner dependent on the presence of this conserved CGACTC motif containing a methylation site.

Since cell division is inhibited in both FtsZ-depleted cells and MipZ-depleted cells (Wang *et al*., 2001; Thanbichler and Shapiro, 2006), we considered that CcrM-mediated methylation might activate *ftsZ* and/or *mipZ* expression in *C. crescentus*. To get a first indication, we compared, by immunoblotting, the intracellular concentrations of FtsZ and MipZ in cell extracts from the wild-type and Δ*ccrM* strains. We found that Δ*ccrM* cells accumulated approximately fivefold and twofold less FtsZ and MipZ, respectively, than wild-type cells when cultivated in minimal medium (Fig. 2B). In rich medium, FtsZ levels were even more dramatically reduced in Δ*ccrM* cells, compared with wild-type cells (Fig. S5). We then compared the mRNA levels of *ftsZ* and *mipZ* in wild-type and Δ*ccrM* cells cultivated in minimal medium by quantitative real-time PCR. We found that *ftsZ* and *mipZ* mRNAs were approximately fourfold and fivefold less abundant in Δ*ccrM* cells than in wild-type cells respectively (Fig. 2C). We concluded that DNA methylation by CcrM promotes the accumulation of the *ftsZ* and *mipZ* mRNAs and FtsZ and MipZ proteins. Insufficient levels of FtsZ and/or MipZ then provided a potential explanation for the cell division defect observed in cells lacking CcrM.

### Expressing *ftsZ* from an inducible promoter strongly attenuates the cell-division defect of cells lacking CcrM cultivated in rich medium

Optimal intracellular levels of FtsZ and MipZ proteins are needed for normal cell division in *C. crescentus* (Thanbichler and Shapiro, 2006). Since FtsZ levels were more affected than MipZ levels in Δ*ccrM* cells (Fig. 2B), we suspected that the cell division defect of Δ*ccrM* cells was more likely due to a lack of FtsZ, than to a lack of MipZ. Populations of cells accumulating limiting amounts of MipZ were shown to contain frequent mini-cells, originating from the assembly of the divisome at the wrong position along the cell axis (Thanbichler and Shapiro, 2006). Consistent with our hypothesis, we did not observe mini-cells in Δ*ccrM* cultures (Fig. 1A). We also observed that fluorescently tagged FtsZ molecules still formed regular fluorescent foci, usually one to three, along the axis of filamentous Δ*ccrM* cells (Fig. S6), indicating that the spatial regulation of the assembly of the Z-ring is not significantly affected in cells that lack CcrM, unlike what was previously observed for filamentous cells depleted for MipZ (Thanbichler and Shapiro, 2006). We conclude that the levels of MipZ in Δ*ccrM* cells are most likely sufficient to ensure the main function of the protein during cell division.

**Fig. 3:**
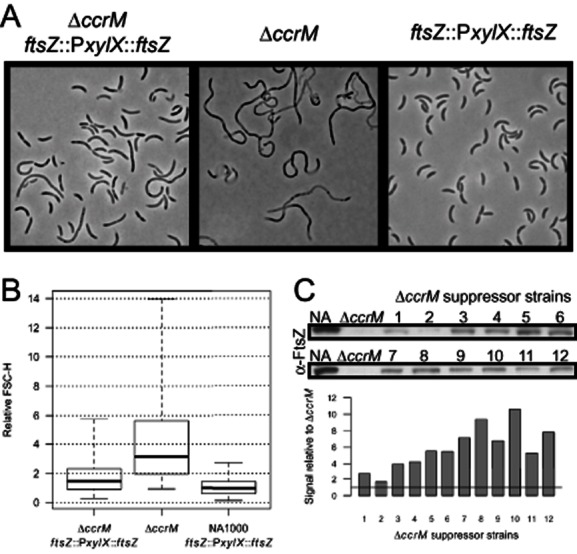
Δ*ccrM* cells expressing *ftsZ* from an inducible promoter are less elongated than Δ*ccrM* cells. A. Phase-contrast microscopy images of JC1149 (Δ*ccrM*), JC948 (Δ*ccrM ftsZ*::P*xylX*::*ftsZ*) and YB1585 (*ftsZ*::P*xylX*::*ftsZ*) cells grown to exponential phase in PYE medium supplemented with 0.3% xylose (PYEX) to induce *ftsZ* expression. B. Cell size distributions in JC1149, JC948 and YB1585 populations cultivated to exponential phase in PYEX medium, as determined by flow cytometry. Forward scattering values (FSC-H) were used as an estimate of cell sizes. The median is shown as a bold line; the limits of the box are the first and the third quartile; the whiskers represent the 5% and 95% quantiles. A total of 20 000 cells were analysed for each population. C. Immunoblot analysis using FtsZ antibodies (Radhakrishnan *et al*., 2010) to estimate the intracellular levels of FtsZ in spontaneous suppressors of the Δ*ccrM* strain (JC1149) cultivated at 22°C in rich medium. Suppressor strains used to prepare cell extracts were JC1228 (1), JC1229 (2), JC1232 (3), JC1233 (4), JC1230 (5), JC1231 (6), JC1224 (7), JC1225 (8), JC1226 (9), JC1227 (10), JC1223 (11) and JC1222 (12). Below the images of the immunoblots is a graph corresponding to the relative FtsZ protein levels: the signal of the immunoblot images was quantified and normalized using an image of a Coomassie blue-stained SDS-PAGE gel prepared using the same cell extracts. Results were further normalized so that estimated FtsZ levels were equivalent to 1 for the Δ*ccrM* strain (straight line in the graph).

If the quantity of FtsZ is the main factor limiting normal cell division in Δ*ccrM* cells, an artificial expression of *ftsZ* in these cells should complement the cell division defect seen in rich medium. To test this prediction, we transduced the Δ*ccrM* mutation into strain YB1585 (Wang *et al*., 2001), expressing *ftsZ* under the control of the xylose-inducible *xylX* promoter (as the only functional copy of *ftsZ*) and into the wild-type strain as a control. We observed that the transduction efficiency on rich medium supplemented with xylose, was much higher when using the YB1585 strain than the wild-type strain as recipient strain (Fig. S7). This first observation suggested that the artificial expression of *ftsZ* may enhance the viability of Δ*ccrM* cells in rich medium. By phase-contrast microcopy, we observed that these Δ*ccrM ftsZ*::P*xylX*::*ftsZ* cells were much shorter than Δ*ccrM* cells when cultivated in rich medium containing xylose (Fig. 3A and B). Flow cytometry analysis confirmed that less than 5% of these Δ*ccrM ftsZ*::P*xylX*::*ftsZ* cells were more than sixfold longer than the median *ftsZ*::P*xylX*::*ftsZ* control cell, compared with about 25% of the Δ*ccrM* cells (Fig. 3B). We found that the distribution of cell sizes was still broader in the Δ*ccrM ftsZ*::P*xylX*::*ftsZ* population (Fig. 3B) than in wild-type (Fig. 1B) or YB1585 (Fig. 3B) populations cultivated in rich medium, but much narrower than in the Δ*ccrM* population (Fig. 3B). Membrane defects, very frequent in Δ*ccrM* cells, were only rarely observed for these Δ*ccrM ftsZ*::P*xylX*::*ftsZ* cells. Overall, our observations demonstrated that the transcription of *ftsZ* from an inducible promoter promotes cell division and enhances the viability of a *C. crescentus* strain that lacks CcrM in rich medium. These results suggest that the intracellular level of the essential FtsZ protein is too limiting for cell division and for normal cell viability in cells lacking CcrM cultivated in rich medium.

### Spontaneous suppressors of the Δ*ccrM* strain accumulate more FtsZ than the Δ*ccrM* strain

If the low intracellular concentration of FtsZ is the main reason why Δ*ccrM* cells cannot divide and die in rich medium, one would expect suppressors of the Δ*ccrM* strain to contain a higher intracellular concentration of FtsZ molecules, or more active FtsZ molecules, than the Δ*ccrM* strain. To test this hypothesis, we isolated 12 independent spontaneous suppressors of the Δ*ccrM* strain when cultivated in rich medium. The generation time of these suppressor strains was approximately double than that of a wild-type strain cultivated in rich medium at 28°C (data not shown). Under the microscope, all 12 suppressor strains looked much less filamentous, or sometimes only slightly elongated, compared with the original Δ*ccrM* strain cultivated in rich medium at 22°C (Fig. S8) or 28°C. To investigate whether cells from the 12 suppressor strains contained more FtsZ than cells from the Δ*ccrM* strain, we performed an immunoblot analysis using extracts from cells grown in rich medium at 22°C, a condition where the Δ*ccrM* cells are very elongated but still viable. We found that FtsZ levels were higher in each suppressor strain, compared with the Δ*ccrM* strain (Fig. 3C). Since all the suppressor strains that we isolated accumulated more FtsZ than the Δ*ccrM* strain, we concluded that insufficient quantities of FtsZ are the main burden on the fitness of Δ*ccrM* cells when cultivated in rich medium. Thus, one of the most important functions of CcrM in *C. crescentus* is to promote FtsZ accumulation to support cell division, especially in rich medium.

Interestingly, the intracellular levels of MipZ were also higher in approximately half of the suppressor strains than in the Δ*ccrM* strain (Fig. S9), suggesting that some suppressor mutations that can promote FtsZ accumulation in the Δ*ccrM* strain can also promote MipZ accumulation. This indicates that a shared regulatory pathway may control the intracellular levels of FtsZ and MipZ.

### CcrM promotes *ftsZ* and *mipZ* transcription

**Fig. 4:**
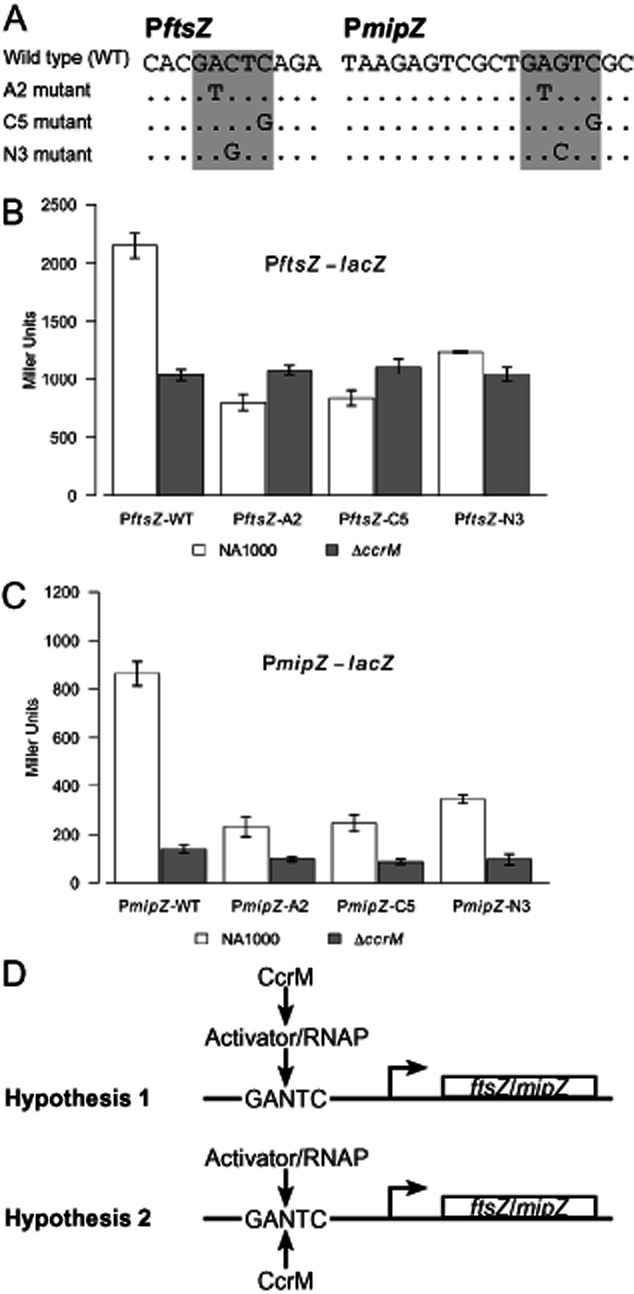
GANTC motifs found in the *ftsZ* and *mipZ* promoter regions are required for maximal *ftsZ* and *mipZ* transcription, in a CcrM-dependent manner. A. Schematics showing the point mutations introduced in the GANTC motifs (highlighted in grey) in the *ftsZ* and *mipZ* promoters fused to the *lacZ* gene in p*lacZ*290 derivatives. The A2 and C5 mutations prevent the methylation of the highlighted motif by CcrM. The N3 mutations do not affect the methylation of the highlighted GANTC motif by CcrM. B. The graph shows the β-galactosidase activities from p*lacZ*290-P*ftsZ*-WT, p*lacZ*290-P*ftsZ*-A5, p*lacZ*290-P*ftsZ*-C5 and p*lacZ*290-P*ftsZ*-N3 in NA1000 (wild-type) and JC1149 (Δ*ccrM*) cells cultivated in exponential phase in M2G. The error bars indicate standard deviations from three independent experiments. C. The graph shows the β-galactosidase activities from p*lacZ*290-P*mipZ*-WT, p*lacZ*290-P*mipZ*-A5, p*lacZ*290-P*mipZ*-C5 and p*lacZ*290-P*mipZ*-N3 in NA1000 and JC1149 (Δ*ccrM*) cells cultivated in exponential phase in M2G. The error bars indicate standard deviations from three independent experiments. D. These schematics depict two hypotheses compatible with the results that are shown in (B) and (C). According to Hypothesis 1, CcrM promotes the transcription of *ftsZ* or *mipZ* indirectly, by enhancing the synthesis or the activity of a direct activator of *ftsZ* or *mipZ* transcription or of an RNAP component. According to Hypothesis 2, CcrM promotes the transcription of *ftsZ* or *mipZ* by directly methylating their promoter regions and thereby promoting the binding or the activity of a direct activator of *ftsZ* or *mipZ* transcription or of an RNAP component.

Since DNA methylation by CcrM can affect the transcription of genes in *C. crescentus*, we hypothesized that the reduced accumulation of *ftsZ* and *mipZ* mRNAs in Δ*ccrM* cells compared with wild-type cells (Fig. 2C) may result from a reduced transcription of the *ftsZ* and *mipZ* genes. To demonstrate that CcrM regulates the activity of the *ftsZ* and *mipZ* promoters, we used two transcriptional fusions between the *ftsZ* and *mipZ* promoters and the *lacZ* reporter gene on low-copy-number plasmids (p*lacZ*290-P*ftsZ*-WT and p*lacZ*290-P*mipZ*-WT plasmids respectively). We compared the β-galactosidase activities of cell extracts from the wild-type and Δ*ccrM* strains containing these plasmids and cultivated in minimal medium. We found that both promoters were more active in the presence of CcrM: the activity of the *ftsZ* promoter was twofold lower, while the activity of the *mipZ* promoter was more than fourfold lower, in the Δ*ccrM* strain than in the wild-type strain (Fig. 4B and C). These results demonstrated that CcrM promotes *ftsZ* and *mipZ* transcription, explaining why FtsZ and MipZ levels were lower in the strain lacking CcrM (Fig. 2B). They were nevertheless not sufficient to determine if CcrM affected the expression of a regulator of *ftsZ* and *mipZ* transcription or if CcrM promoted *ftsZ* and *mipZ* transcription by directly methylating their promoter regions.

Interestingly, the transcription of *ftsZ* and *mipZ* is regulated by the DnaA and CtrA master regulators (Kelly *et al*., 1998; Laub *et al*., 2002; Hottes *et al*., 2005; McGrath *et al*., 2007; Fernandez-Fernandez *et al*., 2011), which are controlled by CcrM-mediated DNA methylation (Reisenauer and Shapiro, 2002; Collier *et al*., 2007). We therefore considered that CcrM may promote *ftsZ* and *mipZ* transcription indirectly through an effect on the expression of their two regulators DnaA and CtrA. To test this possibility for the *ftsZ* promoter, we constructed a mutant *ftsZ* promoter that lacks its DnaA and CtrA binding sites and fused it to the *lacZ* gene to compare its activity in wild-type and Δ*ccrM* cells by β-galactosidase assays. We found that this mutant *ftsZ* promoter was less active in Δ*ccrM* cells than in wild-type cells (Fig. S10), similarly to what was observed using the wild-type *ftsZ* promoter. We concluded that CcrM can still promote the activity of the *ftsZ* promoter when it no longer contains CtrA or DnaA binding sites, suggesting that other motifs in the *ftsZ* promoter are required for the direct or indirect control of *ftsZ* transcription by CcrM.

### The conserved CGACTC motif in the *ftsZ* and *mipZ* promoter regions is required for the efficient transcription of *ftsZ* and *mipZ*

The presence of a shared and conserved CGACTC motif in the promoters of *ftsZ* and *mipZ* suggested that it may be an important promoter element involved in the co-regulation of *ftsZ* and *mipZ* transcription. To test this possibility, we created three variants of each promoter containing point mutations in the CGACTC motif (Fig. 4A), cloned them into the p*lacZ*290 vector and introduced them into the wild-type strain, to compare their activities with the activities of the wild-type promoters. We found that each point mutation strongly decreased the activities of the *ftsZ* and *mipZ* promoters in the wild-type strain (Fig. 4B and C). These results show that the integrity of the conserved CGACTC motif found in the *ftsZ* and *mipZ* promoter regions is required for their maximal activity, suggesting that a transcriptional activator or an RNAP component binds to this motif on each promoter (Fig. 4D). Mutant promoters carrying mutations that did not remove the GANTC methylation site (N3) however appeared significantly more active than un-methylatable mutant promoters (Fig. 4B and C). This last observation is compatible with a direct involvement of DNA methylation in the regulation of *ftsZ* and *mipZ* transcription.

### The conserved CGACTC motif in the *ftsZ* promoter is required for CcrM to promote *ftsZ* transcription

DNA methylation by CcrM (Fig. 2) and the integrity of the conserved CGACTC motif in the *ftsZ* and *mipZ* promoter regions (Fig. 4) are both required for the efficient transcription of *ftsZ* and *mipZ*. If CcrM stimulates the activity of the *ftsZ* and *mipZ* promoters through their conserved CGACTC motif (Fig. 4D), the effect of CcrM on *ftsZ* and *mipZ* transcription is expected to be dependent on the presence of an intact CGACTC motif. To test this possibility, we compared the activity of each mutant *ftsZ* and *mipZ* promoter in the wild-type and in the Δ*ccrM* strains by β-galactosidase assays (Fig. 4B and C).

Using the mutant *ftsZ* promoters, we observed that their activities were not strongly decreased in Δ*ccrM* cells compared with wild-type cells (Fig. 4B), showing that CcrM is dependent on an intact CGACTC motif in the *ftsZ* promoter to stimulate *ftsZ* transcription. Interestingly, all four *ftsZ* promoter variants, including the wild-type promoter, had the same activity in Δ*ccrM* cells (Fig. 4B), indicating that the activatory effect of the CGACTC motif in the *ftsZ* promoter is dependent on the presence of the CcrM methyltransferase.

Using the mutant *mipZ* promoters, we observed that their activities were still two- to threefold lower in the Δ*ccrM* strain compared with the wild-type strain, but to a lesser extent compared with the wild-type promoter (more than fivefold decrease in activity) (Fig. 4C). These results show that the CGACTC motif in the *mipZ* promoter region is required for the efficient promotion of *mipZ* transcription by CcrM, although CcrM still significantly promotes *mipZ* transcription in a manner that does not involve this motif. It is therefore likely that CcrM plays a dual role in the control of *mipZ* expression, by regulating *mipZ* transcription not only through this conserved CGACTC motif, but also independently. Another promoter element that might be involved is the second GANTC methylation site that is found near the CGACTC motif (Fig. 2A).

All together, our findings indicate that the effect of CcrM on *ftsZ* and *mipZ* transcription is at least partially dependent on the presence of the CGACTC motif in the *ftsZ* and *mipZ* promoters.

### The hemi-methylation of GANTC sites in modified *ftsZ* and *mipZ* promoters stimulates their activities

We considered that at least two models were consistent with the results shown in Fig. 4. According to the first one, CcrM promotes the expression of a putative transcriptional activator or RNAP component, which requires the integrity of the CGACTC motif, but not necessarily the methylation of its adenine, to activate the transcription of the *ftsZ* and *mipZ* genes (Hypothesis 1 in Fig. 4D). According to the second one, methylation of the GANTC site in the CGACTC motif found in these promoters stimulates the binding or the activity of a transcriptional activator or RNAP component binding to the CGACTC motif (Hypothesis 2 in Fig. 4D). Notably, both hypotheses can be true at the same time.

**Fig. 5:**
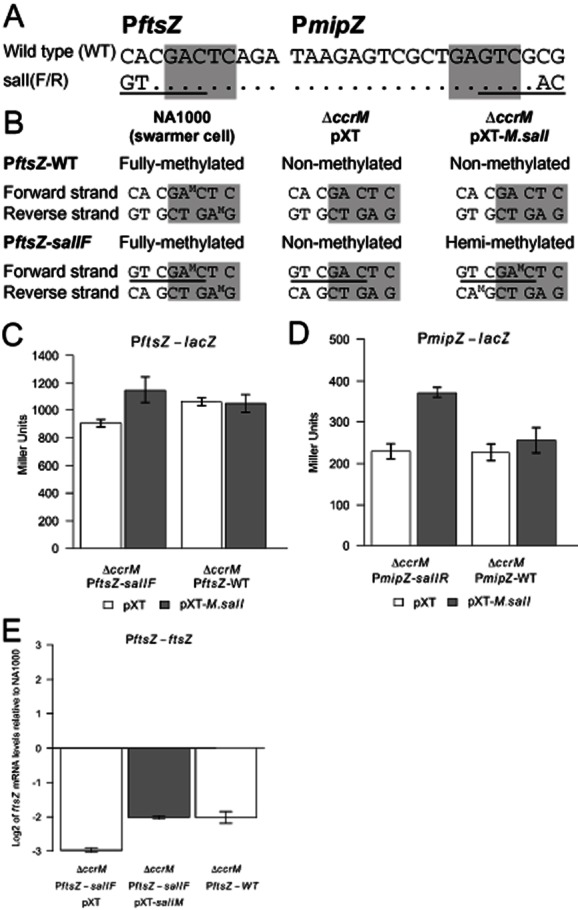
The artificial hemi-methylation of GANTC sites in the *ftsZ*-*salIF* and *mipZ*-*salIR* promoters stimulates their activity in Δ*ccrM* cells. A. Schematics showing the two-nucleotide mutations introduced next to the GANTC motifs (highlighted in grey) in the *ftsZ* and *mipZ* promoters. These mutations create a SalI methylation motif (GTCGAC sites, underlined) on the forward (F) strand of the *ftsZ*-*salIF* promoter and on the reverse (R) strand of the *mipZ*-*salIR* promoter. The GTCGAC motif overlaps but does not disrupts the GANTC motifs (highlighted in grey) from the *ftsZ* and *mipZ* promoters. B. Methylation states of the GANTC motif in P*ftsZ*-*WT* and P*ftsZ*-*salIF* in NA1000 (wild-type) and Δ*ccrM* cells expressing (from pXT-*M.salI*) or not (pXT control vector) the M.SalI methyltransferase (strains JC1084, JC835, JC1147 and JC1127). A^M^ corresponds to methylated adenines. The same principle applies to the *mipZ* promoter variants, except that the reverse strand of the GANTC site is methylated by the M.SalI methyltransferase, instead of the forward strand. C. The graph shows the β-galactosidase activities of extracts of Δ*ccrM* cells containing p*lacZ*290-P*ftsZ*-*salIF* or p*lacZ*290-P*ftsZ*-*WT* and expressing (from pXT-*M.salI*) or not (pXT control vector) the M.SalI methyltransferase (strains JC1147 and JC1127 respectively). Cells were cultivated in exponential phase in M2G medium containing 0.06% of xylose to induce the expression of M.SalI. The error bars indicate standard deviations from six independent experiments. D. The graph shows the β-galactosidase activities from p*lacZ*290-P*mipZ*-*salIR* and p*lacZ*290-P*mipZ*-*WT* in Δ*ccrM* cells expressing (from pXT-*M.salI*) or not (pXT control vector) the M.SalI methyltransferase (strains JC1147 and JC1127). Cells were cultivated in exponential phase in M2G medium containing 0.3% of xylose to induce M.SalI expression. The error bars indicate standard deviations from three independent experiments. E. Quantitative real-time PCR analysis comparing *ftsZ* mRNA levels in NA1000, JC1168 (Δ*ccrM* P*ftsZ*-*salIF*::*ftsZ xylX*::pXT), JC1169 (Δ*ccrM* P*ftsZ*-*salIF*::*ftsZ xylX*::pXT-*M.salI*) and JC1149 (Δ*ccrM*) cells. Cell extracts were prepared from cells cultivated in exponential phase in M2G medium (strains NA1000 and JC1149) or in M2G medium containing 0.06% xylose (strains JC1168 and JC1169). *ftsZ* mRNA levels were quantified using NA1000 as the calibrator and the levels of the *CC_3527* mRNA as an internal reference. The graph shows the log2 of the ratio of *ftsZ* mRNA levels in JC1168, JC1168 or JC1149, compared with the NA1000 strain. Error bars correspond to the standard deviations from three independent biological samples.

To test the second hypothesis *in vivo*, we tried to determine whether the *ftsZ* and *mipZ* promoters were more active when a single adenine in their double-stranded GANTC motifs was methylated (hemi-methylated state) than when the GANTC sites were non-methylated. To do so, we developed a novel method based on the heterologous expression of a M.SalI methyltransferase from *Streptomyces albus* in *C. crescentus*. The M.SalI enzyme methylates adenines on both strands in GTCGAC motifs and protects the DNA from cleavage by the SalI endonuclease (Rodicio *et al*., 1994). We constructed a *C. crescentus* strain that expressed the *S. albus M.salI* gene under the control of the xylose-inducible *xylX* promoter at the native *xylX* chromosomal locus (strain JC1084). We confirmed that M.SalI was active in *C. crescentus* upon xylose addition in the medium, by showing that chromosomes extracted from that strain could not be digested by the SalI endonuclease, whereas chromosomes extracted from a wild-type *C. crescentus* strain could (Fig. S11). We then introduced p*lacZ*290 derivatives carrying wild-type (*WT*) and mutant (*salIF* or *salIR*) *ftsZ* and *mipZ* promoters fused to the *lacZ* gene into this strain and into control strains (Figs S12 and S13). As expected, we observed that the activity of the wild-type *ftsZ* and *mipZ* promoters that cannot be methylated by M.SalI was not significantly affected by the expression of the M.SalI enzyme (Fig. 5C and D, here in a Δ*ccrM* background). The mutations of two non-conserved nucleotides that we engineered in the *ftsZ-salIF* and *mipZ-salIR* promoter variants created GTCGAC SalI motifs (Fig. 5A and B). In each case, the GTCGAC SalI motif overlapped the native GACTC site in the *ftsZ* and *mipZ* promoters, so that the adenine shared by both motifs could be methylated by M.SalI or by the CcrM methyltransferases. Importantly, the CcrM enzyme methylates the adenines on both DNA strands in GACTC motifs (fully methylated GANTC sites), while the M.SalI enzyme methylates the adenine from only one strand in the GACTC motifs in the *ftsZ-salIF* and *mipZ-salIR* promoters (hemi-methylated GANTC sites) (Fig. 5B). Noteworthy, the *ftsZ-salIF* and *mipZ-salIR* promoter regions are still methylated on their two DNA strands in the presence of the M.SalI enzyme: a second adenine that belongs to the M.SalI site is also methylated (Fig. 5B). We compared the activities of the *ftsZ-salIF* and *mipZ-salIR* promoters in Δ*ccrM* cells expressing or not the M.SalI methyltransferase, to see if the methylation state of these promoters directly affected their activities. We found that the *mipZ-salIR* promoter was ∼ 1.5-fold more active in Δ*ccrM* cells when the M.SalI methyltransferase was expressed than when it was not (Fig. 5D). This result shows that the *mipZ-salIR* promoter is more active when its GANTC site is hemi-methylated, than when it is non-methylated. As for the *ftsZ-salIF* promoter, we found by β-galactosidase assays that it was ∼ 1.25-fold more active in Δ*ccrM* cells when the M.SalI methyltransferase was expressed than when it was not (Fig. 5C). To confirm this result, we constructed a Δ*ccrM* strain expressing M.SalI in which the mutant *ftsZ-salIF* promoter drives the expression of the native *ftsZ* gene on the chromosome (Fig. 5A). We measured *ftsZ* mRNA levels in these cells by real-time quantitative PCR and found that they were 1.8-fold more abundant when the M.SalI methyltransferase was expressed than when it was not (Fig. 5E). This result confirms that the *ftsZ-salIF* promoter is more active when its GANTC site is hemi-methylated than when it is non-methylated. All together these data suggest that the native *ftsZ* and *mipZ* promoters may be more active when their conserved GANTC sites are in a hemi-methylated state than when they are in a non-methylated state. This supports our hypothesis 2 (Fig. 4D), according to which the methylation of the conserved GANTC site in the *ftsZ* and *mipZ* promoter regions activates *ftsZ* and *mipZ* transcription.

### The *ftsZ* promoter appears more active when its GANTC site is fully rather than hemi-methylated

The M.SalI-based method that we developed enabled us to show that the *ftsZ* and *mipZ* promoters were more active when their GANTC sites were hemi-methylated rather than non-methylated, but it was not sufficient to test whether there was a difference in activity between hemi-methylated and fully methylated states.

**Fig. 6:**
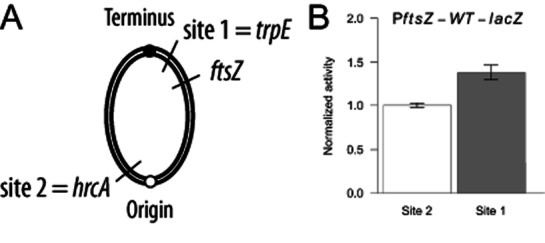
Activity of the *ftsZ* promoter integrated at different chromosomal loci. A. Schematic of the *C. crescentus* chromosome showing the positions of the origin, the terminus and the *ftsZ*, *trpE* (site 1) and *hrcA* (site 2) genes. B. Normalized activity of the P*ftsZ*-WT promoter at site 1 and site 2. β-Galactosidase activities from P*ftsZ*-WT-*lacZ* and P*ftsZ*-C5-*lacZ* reporters in strains JC1269 (P*ftsZ*-WT-*lacZ* at site 1), JC1271 (P*ftsZ*-WT-*lacZ* at site 2), JC1270 (P*ftsZ*-C5-*lacZ* at site 1) and JC1272 (P*ftsZ*-C5-*lacZ* at site 2) cultivated in exponential phase in PYE medium were measured. The activities of P*ftsZ*-WT at site 1 and at site 2 were corrected using the activities of P*ftsZ*-C5 at the respective sites to compensate for effects due to copy number variation, and normalized so that the average activity of P*ftsZ*-WT at site 2 equals 1. The plotted value for P*ftsZ*-WT at site 1 equals (activity of P*ftsZ*-WT at site 1/activity of P*ftsZ*-C5 at site 1)/(activity of P*ftsZ*-WT at site 2/activity of P*ftsZ*-C5 at site 2). Error bars correspond to the standard deviations from three independent biological samples.

**Fig. 7:**
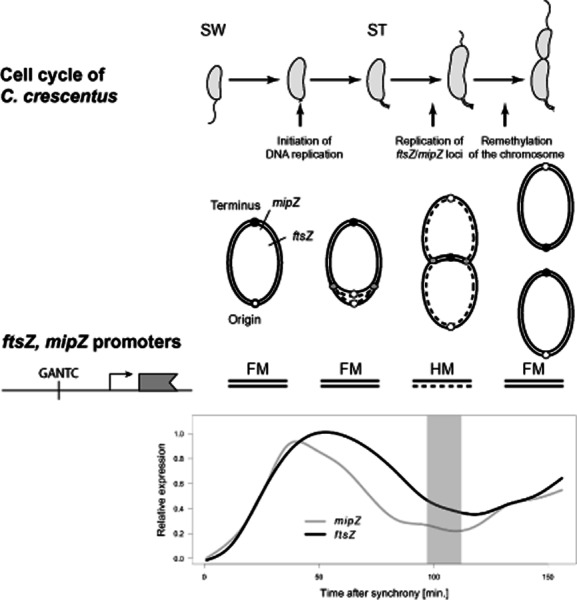
Schematic showing the cell cycle of *C. crescentus*, the methylation state of the chromosome and the temporal variations in *ftsZ* and *mipZ* mRNA levels. SW and ST indicate swarmer and stalked cells respectively. FM and HM indicate fully methylated and hemi-methylated DNA respectively. The relative expression (arbitrary units) of *ftsZ* and *mipZ* as a function of the cell cycle was estimated based on mRNA levels measured from a synchronized population of wild-type swarmer cells cultivated in M2G media (McGrath *et al*., 2007). The grey shade on the graph indicates the approximate period during which the *ftsZ* and *mipZ* promoters are hemi-methylated.

In *C. crescentus*, GANTC sites at loci close to the terminus remain fully methylated during the whole cell cycle whereas GANTC sites at loci close to the origin of replication are in a hemi-methylated state for a long period during the cell cycle (Fig. 7) (Stephens *et al*., 1996; Marczynski, 1999; Collier, 2009). We checked for a possible difference in *ftsZ* promoter activity between the hemi-methylated and the fully methylated states by introducing a P*ftsZ*::*lacZ* transcriptional fusion at two different loci on the *C. crescentus* chromosome: at site 1, located close to the terminus of replication, and at site 2, located near the origin of replication (Fig. 6A). The P*ftsZ*-C5-*lacZ* reporter was also integrated at these two chromosomal sites in two more strains, as controls to measure any change in promoter activity that is not dependent on the GANTC site in the *ftsZ* promoter, such as copy number effects. If the wild-type *ftsZ* promoter is more active when it is fully methylated rather than hemi-methylated, it should be more active when located near the chromosomal terminus than near the origin, as previously shown for other promoters regulated by DNA methylation in *C. crescentus* (Reisenauer and Shapiro, 2002; Collier *et al*., 2006; 2007). When we measured the activity of the methylatable P*ftsZ*-WT promoter at site 1 and 2, and normalized these values to eliminate copy-number effects with values obtained using the un-methylatable P*ftsZ*-C5 promoter, we found that the wild-type *ftsZ* promoter was ∼ 1.4-fold more active when it was integrated at site 1 (near the terminus) than site 2 (near the origin) (Fig. 6B). This difference in activity most probably reflects a difference in the methylation state of the *ftsZ* promoter in most of the cells in the population. This result suggests that the *ftsZ* promoter is more active when it is fully methylated than hemi-methylated. Consistent with this conclusion, we also found that the expression of M.SalI in wild-type *C. crescentus* cells leads to a significant increase in the activity of the P*ftsZ-salIF* promoter (Fig. S14).

Overall, these results strongly support a model according to which the *ftsZ* promoter is more active when it is fully methylated than hemi-methylated.

## Discussion

In this study, we showed that the transcription of *ftsZ* and *mipZ*, encoding two important cell division proteins, is downregulated in the absence of the CcrM DNA methyltransferase in *C. crescentus* (Figs 2 and 4). We presented evidence that links this transcriptional effect to the absence of methylation of adenine residues at specific GANTC motifs in the promoters of these two genes (Figs 5 and 6). In addition, our results suggest that limiting levels of FtsZ are the main cause of the strong cell division defect of Δ*ccrM* cells when cultivated in rich medium (Fig. 3). Thus, CcrM-mediated DNA methylation in *C. crescentus* is not only important to modulate the expression of the DnaA and CtrA master regulators of the cell cycle, as previously described (Reisenauer and Shapiro, 2002; Collier *et al*., 2007; Collier, 2009), but also for the activation of the expression of a minimum of two other genes required for cell division (this work). FtsZ is conserved in all *Alphaproteobacteria*, potentially providing an explanation for the phylogenetic conservation of the *ccrM* gene in at least some of them.

### Model for the activation of *ftsZ* and *mipZ* transcription by DNA methylation

Our targeted mutagenesis experiments showed that the GANTC site found in a conserved CGACTC motif in the *ftsZ* and *mipZ* promoters is required to mediate the effect of CcrM on *ftsZ* and *mipZ* transcription in *C. crescentus* (Fig. 4). Since this motif (Fig. 2A) is between the −35 and the −10 positions relative to the transcriptional start site of the *ftsZ* promoter, and close to the transcriptional start site of the *mipZ* promoter, it is probably a binding site for a transcriptional regulator or for a component of the RNAP complex. Consistent with this, we showed that the activity of these promoters is strongly decreased when a mutation in this motif is introduced, independently of their capacities to be methylated by CcrM (Fig. 4). We considered that the binding of the transcriptional regulator or RNAP component that controls *ftsZ* and *mipZ* expression may be influenced by the methylation state of the CGACTC motif in the *ftsZ* and *mipZ* promoter regions. Indeed, methylated adenines are thought to change the structure of the DNA and can thereby modulate DNA–protein interactions (Wion and Casadesus, 2006). Supporting the idea that *ftsZ* and *mipZ* transcription are more efficiently activated by the regulator or RNAP component when the GANTC motif in their promoter is hemi-methylated than when it is non-methylated, we showed that *ftsZ*-*salIF* and *mipZ*-*salIR* promoters with GANTC motifs that were artificially hemi-methylated by the M.SalI enzyme were more active than when they were non-methylated (Fig. 5). This suggests that DNA methylation contributes to the activation of *ftsZ* and *mipZ* promoters by promoting the binding or the activity of the putative methylation-sensitive transcriptional regulator or RNAP component at these promoters (Fig. 4D, Hypothesis 2). This model does not exclude the possibility that CcrM may, in addition, affect *ftsZ* and *mipZ* promoters in an indirect manner, through the regulation of other genes that may encode regulators of *ftsZ* or *mipZ*.

To compare the efficiency of *ftsZ* transcription when the *ftsZ* promoter is hemi- or fully methylated, we compared its activity when located at two opposite positions on the chromosome. Chromosomal positioning influences the time when a locus is replicated, and thereby also the duration of the period when a locus stays hemi-metylated during the *C. crescentus* cell cycle. In this experiment, the methylation state of each GANTC site on the chromosome, except the GANTC site in the *ftsZ* promoter, remained unaffected, limiting risks to observe indirect effects that CcrM may have on the regulation of *ftsZ* expression. We found that the *ftsZ* promoter was more active at a position close to the terminus than at a position close to the origin (Fig. 6), strongly suggesting that the *ftsZ* promoter is more active when it is fully methylated rather than hemi-methylated. This experiment also demonstrates again that the location of a gene on a bacterial chromosome can influence its expression (Reisenauer and Shapiro, 2002; Collier *et al*., 2007; Collier, 2009).

### The co-regulation of *ftsZ* and *mipZ* transcription during the cell cycle

It was previously shown that *ftsZ* transcription is strongly regulated during the *C. crescentus* cell cycle and that it is most efficient in stalked and early pre-divisional cells and least efficient in swarmer and late pre-divisional cells (Kelly *et al*., 1998; McGrath *et al*., 2007) (Fig. 7). Similarly, the *mipZ* mRNA is most abundant in stalked cells and least abundant in pre-divisional cells (Laub *et al*., 2000; McGrath *et al*., 2007) (Fig. 7). These observations, together with our observation that most suppressor mutations in the Δ*ccrM* strain that promote FtsZ accumulation also promote MipZ accumulation (Fig. 3 and Fig. S9), suggest that the expression of *mipZ* and *ftsZ* share common regulatory pathways. Since the *ftsZ* and *mipZ* genes are located next to the terminus of the *C. crescentus* chromosome, their promoter regions will be fully methylated during most of the cell cycle, except for a very short period of time in pre-divisional cells before the expression of *ccrM* (Fig. 7) (Zweiger *et al*., 1994; Marczynski, 1999). According to previously published results, this period of hemi-methylation would correspond to about 15% and 5% of the duration of the cell cycle for *ftsZ* and *mipZ* respectively (Zweiger *et al*., 1994; Marczynski, 1999). If the *ftsZ* and *mipZ* promoters are both less active when they are hemi-methylated than when they are fully methylated, their transient hemi-methylation in pre-divisional cells may momentarily contribute to limiting *ftsZ* and *mipZ* transcription at that time of the cell cycle (Fig. 7). This transient change in the methylation state of the *ftsZ* and *mipZ* promoters can nevertheless not account alone for the drop of *ftsZ* and *mipZ* expression during about half of the duration of the cell cycle (Fig. 7); additional regulatory mechanisms have to be postulated to fully account for the variation of *ftsZ* and *mipZ* transcription during the *C. crescentus* cell cycle. These probably involve DnaA and CtrA (Kelly *et al*., 1998; Laub *et al*., 2002; Hottes *et al*., 2005; Fernandez-Fernandez *et al*., 2011). The putative methylation-dependent transcriptional activator or RNAP component that probably binds to the CGACTC site in the *ftsZ* and *mipZ* promoters could also contribute to their temporal regulation if it is, for example, more abundant or more active in stalked cells than in other cell types (Fig. 7). The co-regulation of *ftsZ* and *mipZ* may contribute to maintaining balanced intracellular levels of the FtsZ cell division protein and of its inhibitor MipZ to promote cell division (Thanbichler and Shapiro, 2006).

### Conservation of the link between DNA methylation and cell division

In *C. crescentus*, CcrM is required to ensure proper cell division and for normal viability in rich medium (Fig. 1 and Stephens *et al*., 1996). Our results indicate that the intracellular concentration of FtsZ is the main limiting factor preventing proper cell division for a *C. crescentus* strain lacking CcrM-dependent methylation (Figs 2 and 3). We hypothesize that this may also be the case in most *Caulobacterales* and *Rhodobacterales*, as a CGACTC motif is often conserved in *ftsZ* promoter regions in these orders (Table S2). In contrast, the essentiality of CcrM in *Rhizobiales* (Wright *et al*., 1997) is most probably not due to the activation of *ftsZ* or *mipZ* transcription by the methylation of their promoter regions by CcrM, since these do not frequently contain GANTC sites. Instead, CcrM could regulate other essential processes such as DNA replication in *Rhizobiales*, as previously demonstrated for the Dam DNA methyltransferase in *V. cholera* (Demarre and Chattoraj, 2010; Koch *et al*., 2010; Val *et al*., 2012).

### How wide is the CcrM regulon in *C. crescentus*?

So far, evidence indicates that the transcription of at least four genes is regulated by CcrM-mediated DNA methylation in *C. crescentus*. These encode the two essential dual function proteins DnaA and CtrA (Reisenauer and Shapiro, 2002; Collier *et al*., 2007), which act as direct regulators of DNA replication and as global transcriptional regulators, and the two FtsZ and MipZ proteins, which are required for cell division (this work). We found that the cell division and the viability defects of Δ*ccrM* cells can be largely compensated by an increase in *ftsZ* expression (Fig. 3), suggesting that the most critical function of CcrM in *C. crescentus* is to promote *ftsZ* transcription. We nevertheless observed that the morphology of the Δ*ccrM* cells expressing *ftsZ* from the xylose-inducible promoter was still not identical to that of wild-type cells (Fig. 3A): these cells were significantly elongated and straighter than wild-type cells, with shorter stalks. These residual phenotypes suggest that more genes involved in cell division, cell curvature or stalk elongation, may be directly or indirectly regulated by DNA methylation in *C. crescentus*. The promoter regions of genes encoding the crescentin and the CTP synthase that influence cell curvature, or encoding the FtsE protein involved in cell division, for example, contain particularly well conserved GANTC sites indicating that the expression of these genes may also be regulated by DNA methylation. The identification of the complete CcrM regulon in *C. crescentus* is an exciting challenge for the future and the viable Δ*ccrM* strains that we isolated (Fig. 3) will be useful tools for these studies, to understand the multiple functions of CcrM in *C. crescentus*.

## Experimental procedures

### Growth conditions

*Caulobacter crescentus* strains were cultivated in peptone yeast extract (PYE) rich medium or in M2 minimal salts plus 0.2% glucose (M2G) minimal medium at 28°C (Ely, 1991), except when indicated otherwise. *Escherichia coli* strains were cultivated in Luria Broth (LB) rich medium. 1.5% agar (A) was added into plates. Antibiotics concentrations used to cultivate *C. crescentus* were the following in μg ml^−1^: oxytetracycline (PYE: 1, PYEA: 2; M2G: 1; M2GA: 2), spectromycin (PYE: 5, PYEA: 5; M2G: 5; M2GA: 10), spectinomycin (PYE: 25, PYEA: 100; M2G: 25; M2GA: 200) and kanamycin (PYE: 5, PYEA: 25; M2G: 5; M2GA: 25). When needed, d-xylose was added at 0.3% final concentration, except when indicated otherwise. All Δ*ccrM* strains, with the exception of strain JC948 and of the Δ*ccrM* strains that accumulated suppressor mutations, were grown on M2GA plates or cultivated in M2G liquid medium with the appropriate antibiotics. Some experiments (immunoblots in Fig. 3C) using Δ*ccrM* cells were carried out in PYE at 22°C with agitation: at this lower temperature, very few cells lyse, but they do have a filamentous morphology (Fig. S8). Strain JC948 was cultivated on PYEA + 0.3% xylose plates or in PYE + 0.3% xylose with the appropriate antibiotics. The Δ*ccrM* strains with suppressor mutations were cultivated on PYEA with the appropriate antibiotics.

### GANTC sites conservation

The degree of conservation of the GANTC sites was determined using the available genomic sequences and annotations of *C. crescentus* NA1000 (NC_011916), *Caulobacter segnis* (NC_014100), *Caulobacter K31* (NC_010333), *Phenylobacter zucineum* (NC_011143), *Brevundimonas subvibrioides* (NC_014375), *Asticcacaulis excentricus* (NC_0148816) and *Maricaulis maris* (NC_008347) from the NCBI FTP server. 5′ UTR sequences were extracted using a home-made Perl program and aligned using the multiple alignment program *muscle*; the alignments were visualized using Jalview or clustalx.

### Microscopy

Microscopy experiments were performed as previously described (Fernandez-Fernandez *et al*., 2011). For live/dead staining procedures, cells were resuspended in 8 mM Mg_2_SO_4_ with 5 μg ml^−1^ 4′,6-diamidino-2-phenylindole (DAPI) and 5 μg ml^−1^ propidium iodide (PI) and visualized with the fluorescence microscope system after a 30 min incubation at room temperature. An RFP filter was used to detect PI and a DAPI filter was used to detect DAPI.

### Flow cytometry analysis

Flow cytometry analysis were performed as previously described (Fernandez-Fernandez *et al*., 2011). Minimum 20 000 cells from each biological sample were stained with Vybrant® DyeCycle™ Orange (Invitrogen, DNA stain) and analysed. Data were collected using the FL-2 fluorescence. Data were analysed and visualized with R [using the ‘prada’ package (Florian Hahne, Wolfgang Huber, Markus Ruschhaupt and Joern Toedling. Prada: data analysis for cell-based functional assays. R package version 1.24.0)]. The forward scattering [FSC] parameter was used to estimate cell sizes.

### Immunoblot analysis

FtsZ and MipZ proteins were resolved on 10% or 12% SDS/PAGE respectively (Sambrook *et al*., 1989). Gels were electrotransferred to a PVDF membrane (Millipore). Immunodetection was performed with polyclonal antibodies. Anti-FtsZ (Mohl *et al*., 2001), anti-FtsZ (Radhakrishnan *et al*., 2010), anti-MipZ (Thanbichler and Shapiro, 2006) and anti-rabbit conjugated to horseradish peroxidase (Sigma Aldrich) sera were diluted 1:4000, 1:30 000, 1:10 000 or 1:10 000 respectively. Chemiluminescence detection, image processing and measurements of relative band intensities were done as previously described (Fernandez-Fernandez *et al*., 2011).

### β-Galactosidase assays

β-Galactosidase assays were carried out using a standard protocol (Miller, 1972). Cells were cultivated to exponential phase in M2G containing oxytetracycline when needed. Promoter activities shown in this study are the averages of at least three biological replicates. The experiments involving the M.SalI methyltransferase were carried out in M2G containing oxytetracycline and 0.3% (for the *mipZ* promoter) or 0.06% (for the *ftsZ* promoter) xylose.

### Quantitative real-time PCR

RNAs were purified from 1 ml of cultures at an OD_660_ of 0.3, pelleted and immediately frozen in liquid nitrogen and stored at −80°C for maximum 2 weeks, using a Trizol (Invitrogen, manufacturer's protocol) extraction, followed by an isopropanol precipitation step, a washing step with 75% ethanol and resuspension into 30 μl of RNase free H_2_O. RNAs were precipitated again in 2 M LiCl at −20°C for 1 h, washed with 75% ethanol and resuspended into 20 μl of RNase-free H_2_O. One microgram of the purified RNA, previously quantified with a Nanodrop Fluorospectrometer and quality-checked on an agarose gel after electrophoresis, was treated with DNase I (Promega) or TURBO™ DNase (Ambion) according to the manufacturer's protocol, checked for the absence of DNA contamination by PCR or qRT-PCR, retrotranscribed with the SuperScript II (Invitrogen) in a 20 μl reaction and treated with RNase H (Invitrogen). For the qRT-PCR, 4 μl of a 1:4 dilution of the cDNA samples from three biological replicates for each strain were used as a template; three technical replicates of each cDNA sample were analysed. For the Fig. 2C, a 20 μl reaction containing 1 μM primers and the KAPA Sybr® FAST ABI Prism qRT-PCR buffer with ROX was used in a Stratagen MX3005P qRT-PCR machine with automated threshold calculation. Cycling: 10 s at 95°C and 30 s at 60°C for 40 cycles. For each pair of primers, the efficiency was calculated on serial dilutions from 10^2^ to 10^8^ copies of template per reaction, the template being a specific PCR product. The internal control used was the *CC_3527* gene (succinate dehydrogenase flavoprotein subunit) for *ftsZ* and *mipZ*. *CC_3527* mRNA levels were shown to be stable in most conditions previously published and they do not change between minimal and rich medium (Hottes *et al*., 2004; McGrath *et al*., 2007). The final ratios are an average of the individual ratios of each of the biological replicates, which were calculated using a formula integrating a correction for the efficiency: Ratio = (E_target_)^ΔCt target (calibrator − sample)^/(E_reference_)^ΔCt reference (calibrator − sample)^, where the target is the tested gene, the reference is *CC_3527*, the calibrator is NA1000, the sample is the mutant strain, E is the efficiency of a primer pair, ΔCt is the difference between the cycle in which there is a significant increase in fluorescence signal above the threshold. For Fig. 5E, a 20 μl reaction containing 1 μM primers and the Qiagen Rotor-Gene SYBR Green were used in a RotorGene Q qRT-PCR machine with automated threshold calculation; cycling: 5 s at 95°C and 10 s at 60°C for 40 cycles; the standard Delta-Delta-Ct algorithm of the Qiagen software was applied to calculate the ratios using *CC_3527* as internal reference and NA1000 as the calibrator. In all cases, dissociation curves and/or a gel electrophoresis of the amplification products were carried out to ensure that no parasitic product was present. When necessary, the significance of the difference in expression was confirmed using a Student's *t*-test.

### Isolation of Δ*ccrM* suppressor strains

Since most Δ*ccrM* cells were still viable on plates, we could not use classical selection procedures on plates to isolate suppressors of the JC1149 strain. Instead, we cultivated three independent colonies of strain JC1149 in M2G media at 28°C. Each overnight culture was used to inoculate four new cultures in PYE media at a final OD_660_ of 0.01. These cultures where cultivated at 28°C until their OD_660_ reached minimum 0.3 and maximum 1.0, before being diluted daily to an OD_660_ of 0.001. These dilutions were repeated for 7 days. In PYE medium Δ*ccrM* cells rapidly accumulated suppressor mutations and gained fitness, thereby becoming predominant in the cell populations over time. At the end of the experiment, cultures were frozen at −80°C. Individual colonies of Δ*ccrM* cells carrying spontaneous suppressor mutations were isolated from the frozen cultures stroked on PYEA plates. One colony isolated from each culture was used for further experiments (strains JC1222 to JC1233), to measure the doubling times of these strains, to analyse their morphology by microscopy and to prepare cell extracts for immunoblot analysis.

### Bacterial strains, plasmids and oligonucleotides

Oligonucleotides used in this study are listed and described in Table S4. Bacterial strains and plasmids used in this study are listed and described in Tables S5 and S6 respectively. Construction of plasmids and strains, including transformation and transduction procedures, are described in *Supporting information*.
