# Supplementary material for: DNA methylation by CcrM activates the transcription of two genes required for the division of Caulobacter crescentus
Source: Mol Microbiol. 2013 Mar 11;88(1):203–18. doi: 10.1111/mmi.12180 (PMC3708114; doi:10.1111/mmi.12180)
Supplement: Supplementary file 1 [file mmi0088-0203-SD1.pdf]

DNA Methylation by CcrM Activates the Transcription of Two Genes Required for the Division of *Caulobacter crescentus*

Diego Gonzalez and Justine Collier

Department of Fundamental Microbiology, Faculty of Biology and Medicine, University of Lausanne, Quartier UNIL/Sorge, Lausanne, CH 1015, Switzerland

# To whom correspondence should be addressed.

E-mail: justine.collier@unil.ch

Telephone: +41-21-692-5610

Fax: +41-21-692-5605

Supporting information

1. Supplementary Figures:

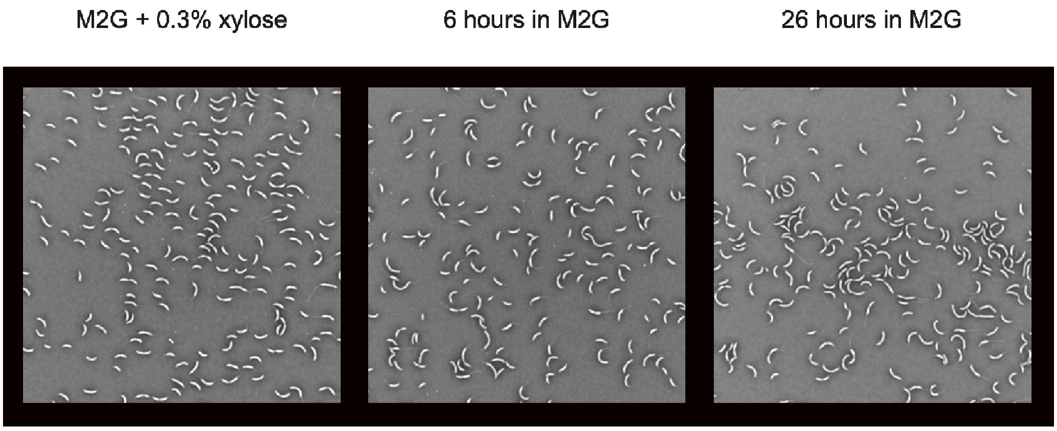

Figure S1: Morphology of cells depleted for CcrM and cultivated in minimal medium. The LS2144 strain (Δ*ccrM*::Ω pCS226), expressing the only copy of *ccrM* from the xylose-inducible *xylX* promoter, was cultivated to exponential phase at 28°C in M2G+0.3% xylose (expression of *ccrM*), and then washed and resuspended in M2G medium (no expression of *ccrM*). Cells were imaged by phase contrast microscopy before the washes and 6 and 26 hours after the washes. The OD_{660nm} continuously increased until stationary phase during growth in M2G medium.

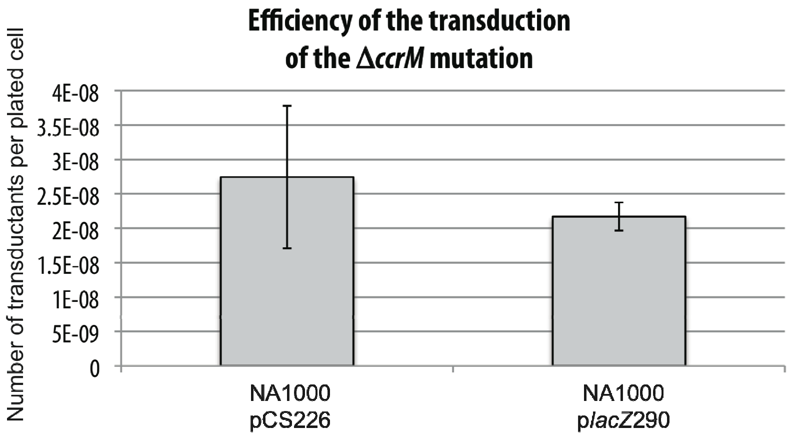

Figure S2: Efficiency of the transduction of the Δ*ccrM*::Ω mutation into cells expressing or not *ccrM* from a xylose-inducible promoter. The Δ*ccrM::*Ω mutation (the Ω cassette renders cells resistant to spectinomycin and spectromycin) was transduced into NA1000 strains containing p*lacZ*290 or pSC226 (expressing *ccrM* from the *xylX* promoter). Before plating on M2GA containing 0.3% xylose, oxytetracyclin, spectinomycin and streptomycin, an aliquot of each culture was sampled and diluted to

the 10^{-4}; 10 and 100 µl were plated on M2GA containing oxytetracyclin to calculate the colony forming units (CFU) in the absence of selection. Transduction frequencies were calculated by dividing the number of colonies obtained on spectinomycin and streptomycin-containing plates by the number of plated CFUs. This experiment was performed in duplicates to evaluate the efficiency of transduction of the Δ*ccrM::*Ω mutation into the complemented and the non-complemented strains.

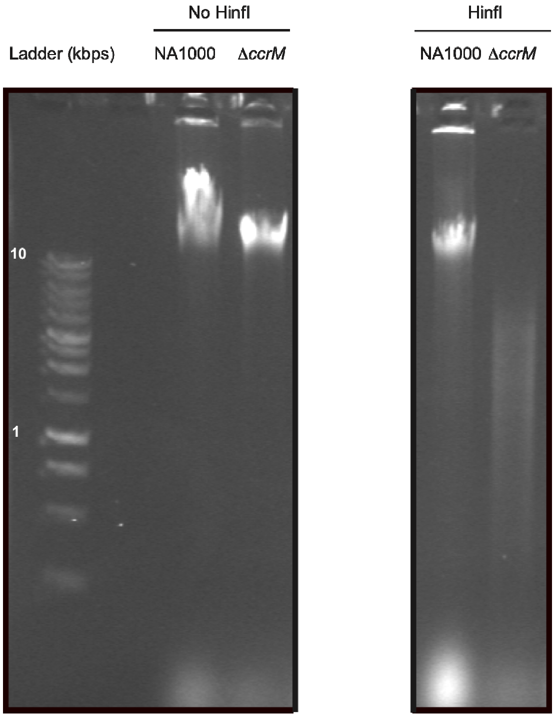

Figure S3: The chromosome of strain JC1149 is sensitive to digestion by the HinfI enzyme. The genomic DNA from strains NA1000 and JC1149 (Δ*ccrM)* cultivated in M2G were extracted using the Quiagen DNeasy kit according to the manufacturer's protocol. 2 µg of genomic DNA were digested with 0.5 µl of HinfI (Promega) enzyme in 20 µl of buffer B. 2 µg of digested and undigested genomic DNA from both strains were subjected to electrophoresis in TAE buffer on a 0.8% agarose gel and visualized under UV light for imaging. This experiment demonstrated that the chromosome of strain JC1149 is not methylated on adenines at GANTC sites, confirming its Δ*ccrM* genotype.

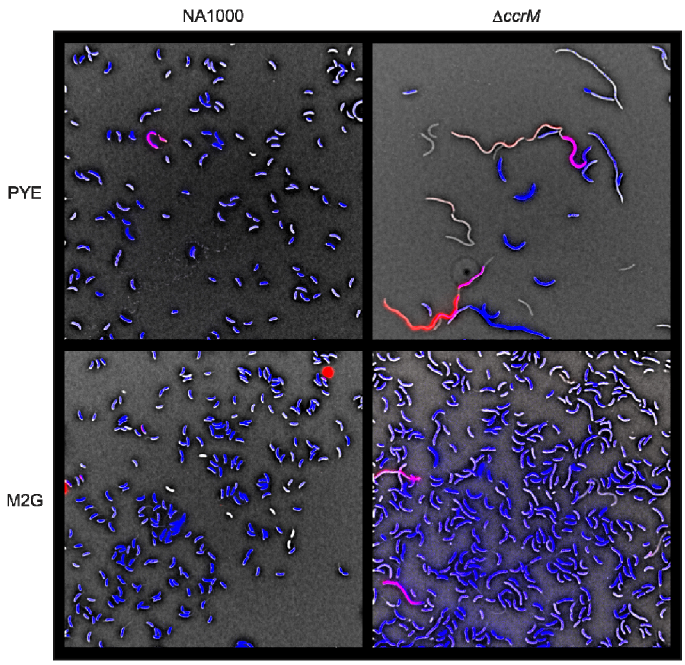

Figure S4: Assay to compare the viability of wild-type and Δ*ccrM* cells in rich and minimal media. NA1000 and JC1149 (Δ*ccrM)* cells were cultivated to exponential phase in PYE or M2G media. Cells were then stained with live/dead staining (DAPI in blue and PI in red) and then visualized by phase contrast and fluorescence microscopy. The figure shows overlaps of phase contrast and fluorescence images. This experiment showed that Δ*ccrM* cells are much more viable in minimal than in rich medium.

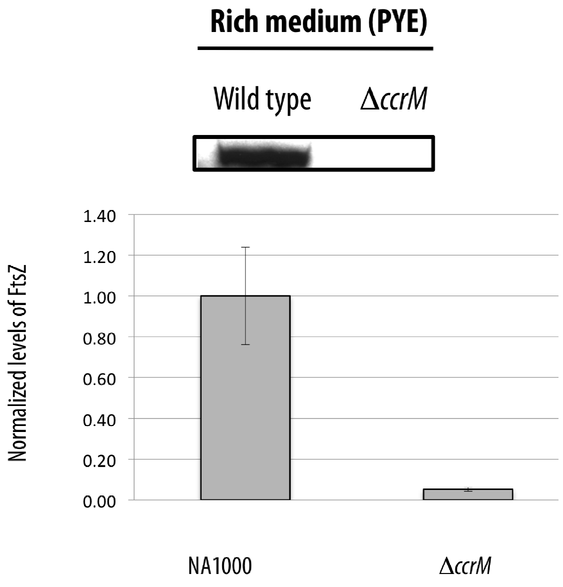

Figure S5: Immunoblot analysis comparing the intracellular levels of FtsZ in wild-type and Δ*ccrM* cells cultivated in rich medium. NA1000 and JC1149 (Δ*ccrM*) cells were cultivated to exponential phase at 28°C in PYE medium. The graphs below the images show relative signal quantifications using images obtained using cell extracts from minimum two independent cultures; the normalization factor is the average of NA1000 signal quantification values for each protein. The OD660 was used to normalize the

global protein content in each cell extract; to compensate for possible biases in OD660 values due to differences in cell shape and length, a stable non-specific protein signal also detected by immunoblot was used as a second normalization factor for the relative quantification of blots. This experiment showed that FtsZ is much less abundant in cells that lack CcrM, than in wild-type cells cultivated in rich medium.

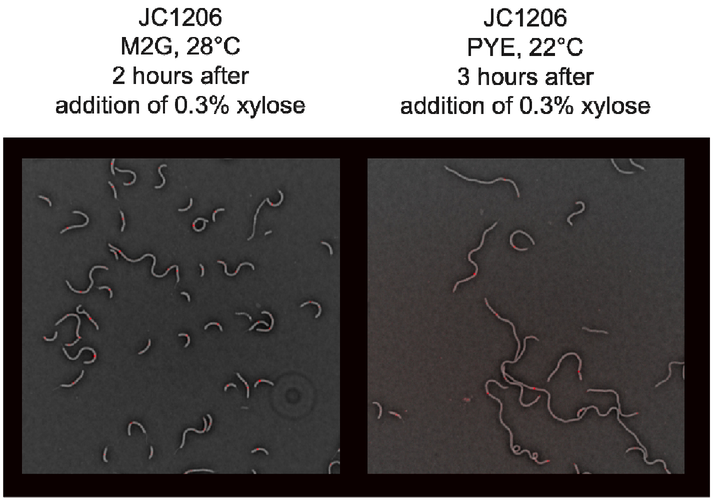

Figure S6: The subcellular localization of FtsZ-YFP in a Δ*ccrM* strain. Strain JC1206 (*xylX*::P*xylX*::*ftsZ-YFP* Δ*ccrM*) was cultivated at 22°C in PYE medium or at 28°C in M2G medium with added 0.3% xylose to induce the expression of *ftsZ-YFP*. Cells were visualized by phase contrast and fluorescence microscopy. The figure shows overlaps of phase contrast and fluorescence images (red colour corresponds to the YFP signal). It is important to note that the FtsZ-YFP fusion protein is not functional for cell division in *C. crescentus*, explaining why the JC1206 cells are very elongated, despite the expression of *ftsZ-yfp* from the *xylX* promoter. This experiment showed that FtsZ-YFP still forms localized fluorescent foci in cells lacking CcrM, suggesting that MipZ levels are not too limiting to control FtsZ localization in these cells.

Strains	Cells per 100 µl (*10^8)	Δ*ccrM*::Ω colonies after a 3 day incubation	Transduction frequency
NA1000 1	1.54	0	0
NA1000 2	1.51	0	0
NA1000 3	1.08	0	0
YB1585 1	1.07	82	7.66355E-08
YB1585 2	1.08	101	9.35185E-08
YB1585 3	0.96	82	8.54167E-08

	**Average transduction frequency**	**Standard deviation**
**NA1000**	0	0
**YB1585**	8.51902E-08	8.44378E-09

Figure S7: Efficiency of the transduction of the Δ*ccrM*::Ω mutation into cells expressing or not *ftsZ* from a xylose-inducible promoter. The Δ*ccrM*::Ω mutation

was transduced into NA1000 and YB1585 (*ftsZ*::P*xyl*X::*ftsZ*) strains. Before plating on PYEA containing 0.3% xylose, spectinomycin and streptomycin, the CFU from an aliquot of each culture was calculated on PYEA plates. Transduction frequencies were calculated by dividing the number of colonies obtained on spectinomycin and streptomycin-containing plates by the CFUs measured in the absence of selection. This experiment was performed in triplicates to evaluate the efficiency of transduction of the Δ*ccrM*::Ω mutation into both strains.

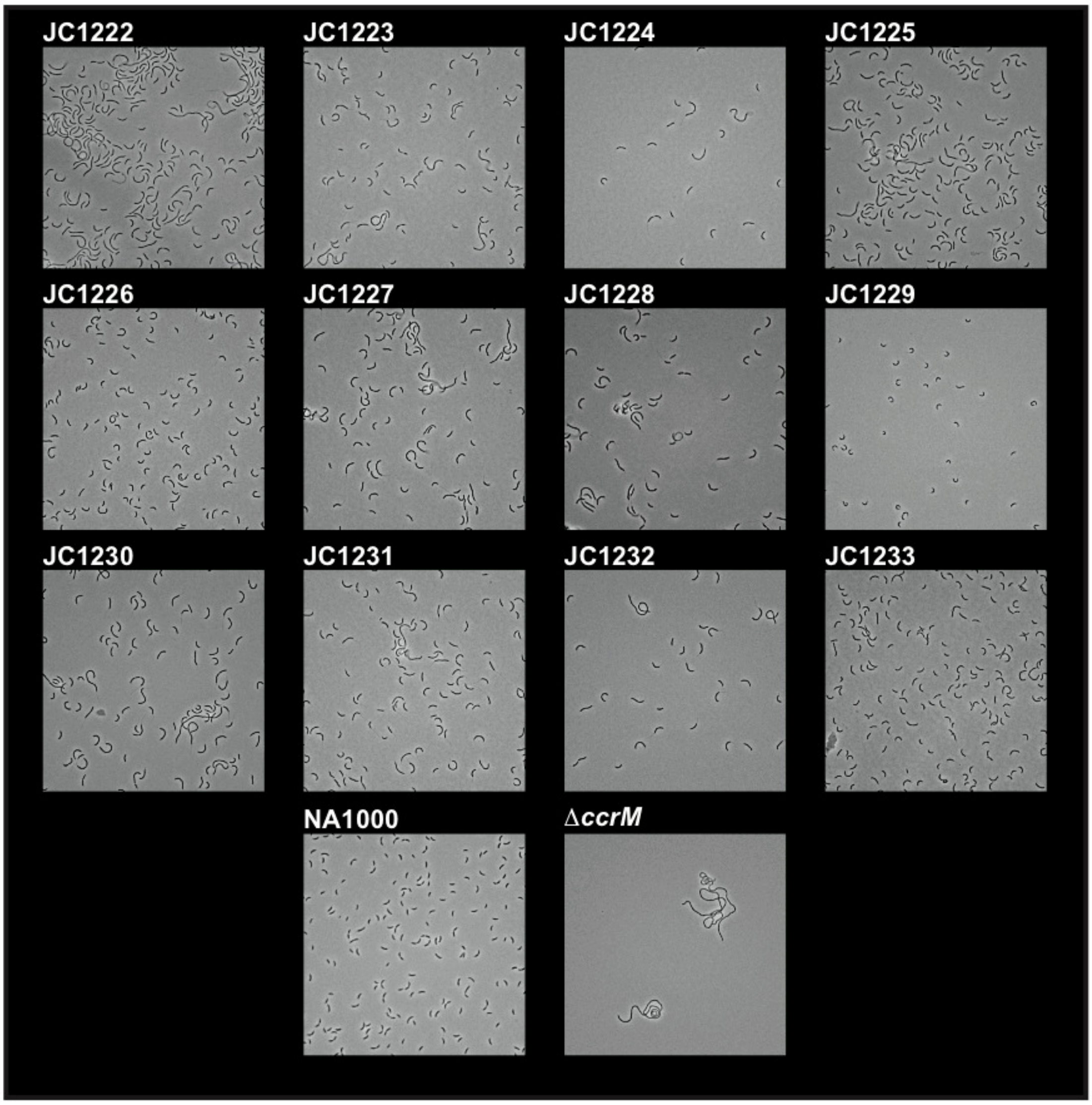

Figure S8: Morphology of cells from the Δ*ccrM* suppressor strains cultivated in rich medium at 22°C. Strains JC1222 to JC1233 (Δ*ccrM* suppressor strains), NA1000 and JC1149 (Δ*ccrM*) were cultivated to exponential phase in PYE medium at 22°C. Cells were then visualized by phase contrast microscopy to compare their morphology. Representative images are shown in this figure. This experiment shows that the 12 Δ*ccrM* suppressor strains that we isolated make cells that are much shorter than Δ*ccrM* cells in these conditions.

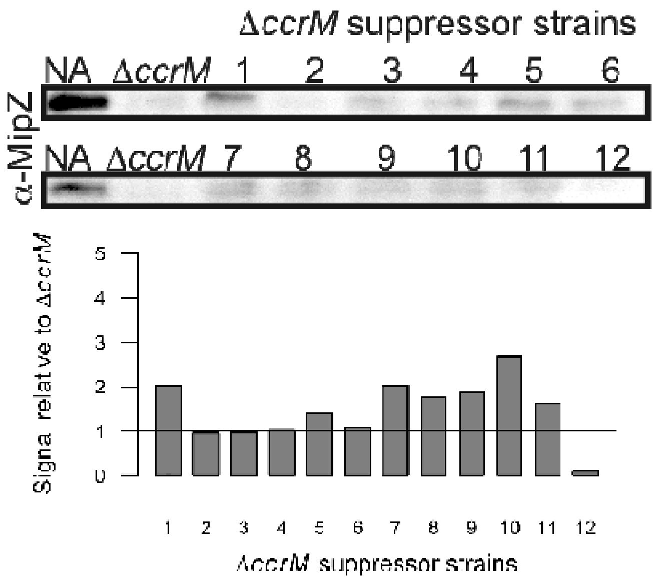

Figure S9: The intracellular levels of MipZ are often higher in the suppressor strains than in the Δ*ccrM* strain. Immunoblot analysis using MipZ antibodies to estimate the intracellular levels of MipZ in spontaneous suppressors of the Δ*ccrM* strain (JC1149) cultivated at 22°C in rich medium. In these growth conditions, MipZ accumulated approximately 7.5-fold more in the wild-type than in the Δ*ccrM* strain. Suppressor strains used to prepare cell extracts were JC1228 (1), JC1229 (2), JC1232 (3), JC1233 (4), JC1230 (5), JC1231 (6), JC1224 (7), JC1225 (8), JC1226 (9), JC1227 (10), JC1223 (11) and JC1222 (12). Below the images of the immunoblots is a graph corresponding to the relative MipZ protein levels: the signal of the immunoblot images was quantified and normalized using an image of a Coomassie blue-stained SDS-PAGE gel prepared using the same cell extracts. Results were further normalized so that estimated MipZ levels were equivalent to 1 for the Δ*ccrM* strain (straight line in the graph).

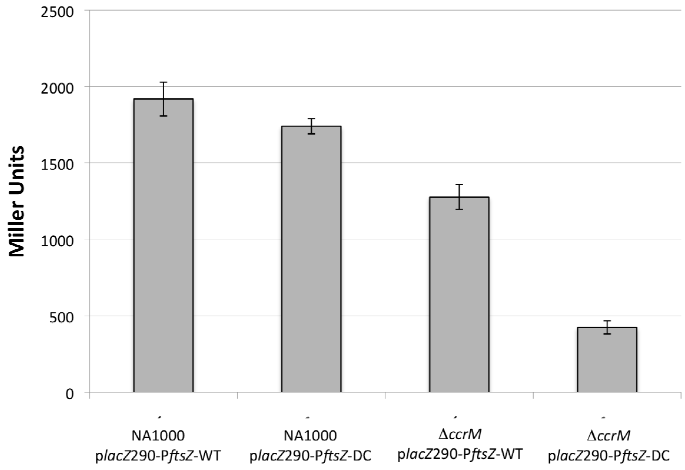

Figure S10: The activity of a *ftsZ* promoter mutated for its CtrA and DnaA binding sites is still stimulated by CcrM. NA1000 and JC1149 (Δ*ccrM*) cells containing the p*lacZ*290-

P*ftsZ*-WT or the p*lacZ*290-P*ftsZ*-DC plasmids were cultivated to exponential phase in M2G. The p*lacZ*290-P*ftsZ*-DC plasmid carries a mutant *ftsZ* promoter with mutations in the CtrA binding site and in the two putative DnaA binding sites. The graph shows the ?-galactosidase activities measured for each strain. The error bars indicate standard deviations from three independent experiments.

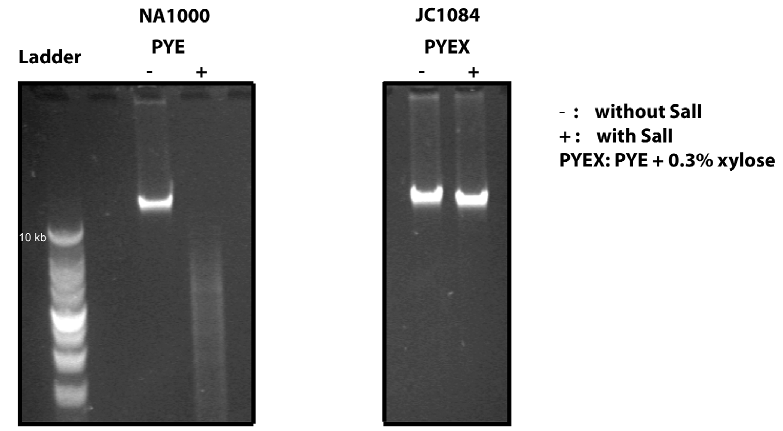

Figure S11: The chromosome of the strain expressing the M.SalI methyltransferase is protected from SalI-mediated digestion. Strains NA1000 and JC1084 (*xylX*::pXT-*M.salI*) were cultivated in PYE and PYE + xylose 0.3%, respectively. Their chromosomal DNA was then extracted and analyzed by gel electrophoresis before and after digestion with the SalI enzyme. This experiment demonstrated that the chromosome of the strain that expresses the M.SalI methyltransferase is methylated by M.SalI, since it is protected from SalI-mediated digestion.

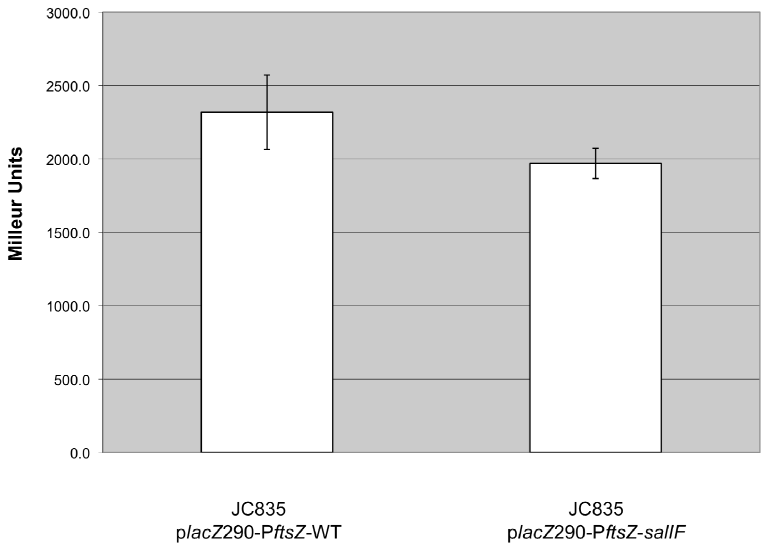

Figure S12: Comparison between the activities of the *ftsZ-WT* and the *ftsZ-salIF* promoters in wild-type cells. JC835 cells (*xylX*::pXT) containing the p*lacZ*290-P*ftsZ*-WT or the p*lacZ*290-P*ftsZ*-*salIF* plasmids were cultivated to exponential phase in M2G+0.3%

xylose. The graph shows the β-galactosidase activities measured for each strain. The error bars indicate standard deviations from three independent experiments.

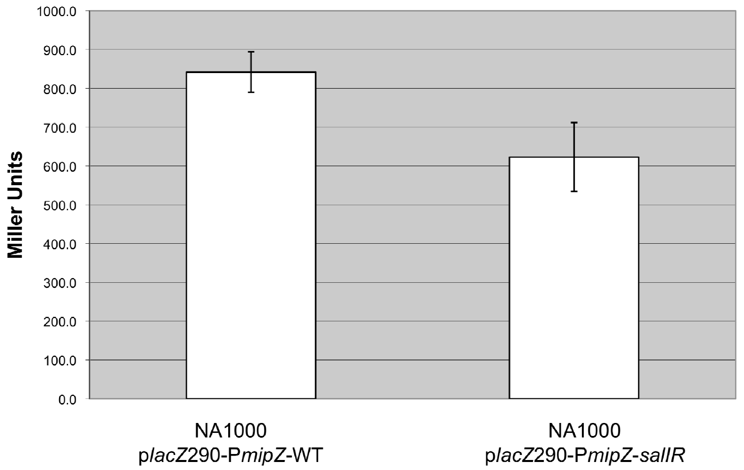

Figure S13: Comparison between the activities of the *mipZ-WT* and the *mipZ-salIR* promoters in wild-type cells. NA1000 cells containing the p*lacZ*290-P*mipZ*-WT or the p*lacZ*290-P*mipZ-salIR* plasmids were cultivated to exponential phase in M2G. The graph shows the β-galactosidase activities measured for each strain. The error bars indicate standard deviations from three independent experiments.

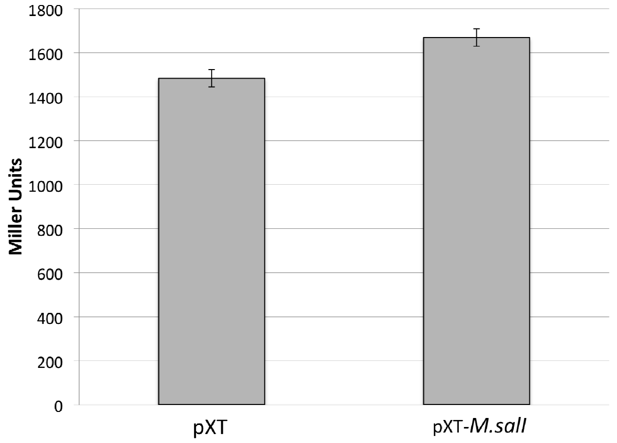

Figure S14: Expression of M.SalI slightly promotes the activity of the *ftsZ-salIF* promoter in wild-type cells. The graph shows the β-galactosidase activities of extracts of wild-type cells containing p*lacZ*290-P*ftsZ-salIF* and expressing (from pXT-*M.salI*) or not (pXT control vector) the M.SalI methyltransferase (strains JC1084 and JC835, respectively). Cells were cultivated in exponential phase in PYE medium containing 0.06% of xylose to induce the expression of M.SalI. The error bars indicate standard deviations from three independent experiments.

In wild-type cells that do not express M.SalI, the GANTC site in the *ftsZ-salIF* promoter on a p*lacZ*290 derivative should be hemi-methylated in ~50% of the cells in a mixed population (Zweiger *et al.*, 1994). When M.SalI is expressed, half of these hemi-methylated GANTC sites (in ~25% of the cells in a population) will be immediately methylated by the M.SalI enzyme (when the new DNA strand contains the adenine that can be methylated by M.SalI). The results indicated that the *ftsZ-salIF* promoter is slightly (12%) more active in a mixed population of wild-type cells expressing the M.SalI methylase than not expressing it. Considering that a change in methylation state due to M.SalI is expected to take place in only 25% of the cells, this difference may be considered as biologically significant. This observation indicated that the *ftsZ* promoter may be more active when its GANTC site is fully-methylated rather than hemi-methylated.

	Caulobacter crescentus NA1000	*Caulobacter segnis ATCC 21756 uid41709*	*Caulobacter K31*	*Phenylobacterium zucineum HLK1*	*Brevundimonas subvibrioides ATCC 15264 uid42117*	*Asticcacaulis excentricus CB 48 uid55641*	*Maricaulis maris MCS10*	Total organisms			
CCNA_00099	0	0	0	0	0	0	122	1	CCNA_00099	CC_0100	fzlC
CCNA_01552	0	12	0	160	152	190	0	4	CCNA_01552	CC_1485	tipN
CCNA_01612	148	96	138	83	0	165	12	6	CCNA_01612	CC_1543	mreB
CCNA_01797	21	21	7	10	0	0	NA	4	CCNA_01797	CC_1725	ftsB
CCNA_02075	148	47	0	30	0	0	0	3	CCNA_02075	CC_1996	dipM
CCNA_02086	51	47	12	49	31	NA	NA	5	CCNA_02086	CC_2007	ftsN
**CCNA_02246**	**25**	**26**	**27**	**26**	**32**	**21**	**57**	**7**	**CCNA_0224[illegible]**	**CC_2165**	**mipZ**
**CCNA_02299**	**74**	**113**	**49**	**120**	**130**	**90**	**78**	**7**	**CCNA 0229[illegible]**	**CC_2216**	**ftsE**
CCNA_02300	0	0	0	0	0	133	NA	1	CCNA_02300	CC_2217	ftsX
**CCNA_02623**	**131**	**131**	**60**	**124**	**129**	**145**	**39**	**7**	**CCNA 0262[illegible]**	**CC_2540**	**ftsZ**
CCNA_02624	138	138	139	136	0	0	0	4	CCNA_02624	CC_2541	ftsA
CCNA_02625	0	42	159	0	0	0	94	3	CCNA_02625	CC_2542	ftsQ
CCNA_02634	0	0	0	0	61	0	0	1	CCNA_02634	CC_2551	murG
CCNA_02635	10	10	0	0	0	67	0	3	CCNA_02635	CC_2552	ftsW
CCNA_02643	0	NA	0	0	0	0	0	0	CCNA_02643	CC_2560	ftsI
CCNA_02644	0	0	NA	NA	NA	NA	NA	0	CCNA_02644	CC_2561	ftsL
CCNA_03337	109	0	0	55	0	0	0	2	CCNA_03337	CC_3229	pal
CCNA_03338	156	159	0	0	0	0	0	2	CCNA_03338	CC_3230	tolB
CCNA_03339	0	0	0	0	NA	NA	NA	0	CCNA_03339	CC_3231	tolA
CCNA_03340	0	0	0	0	0	0	0	0	CCNA_03340	CC_3232	tolR
CCNA_03341	0	0	0	0	7	0	78	2	CCNA_03341	CC_3233	tolQ
CCNA_03356	19	19	0	0	0	153	0	3	CCNA_03356	CC_3247	zapA
CCNA_03691	112	0	0	87	0	25	NA	3	CCNA_03691	CC_3576	kidO
CCNA_03754	0	0	0	28	0	0	195	2	CCNA_03754	CC_3639	fzlA
CCNA_03819	0	108	92	NA	0	0	0	2	CCNA_03819	CC_3704	ftsK

NA	No homologous protein
0	No GANTC
Number	Position of a GANTC upstream the ATG (bps)

Table S1: Conservation of GANTC sites in promoter regions of genes encoding proteins directly or indirectly involved in cell division. GANTC sites were searched in the 200 base pairs (bps) upstream of the coding sequence of the best reciprocal protein blast hit for all known components of the divisome in the *C. crescentus* NA1000 strain according to (Goley *et al.*, 2011), in *Caulobacter segnis* ATCC 21756 uid41709 (NC_014100), *Caulobacter* K31 (NC_010338), *Phenylobacterium zucineum* HLK1 (NC_011144), *Brevundimonas subvibrioides* ATCC 15264 uid42117 (NC_014375), *Asticcacaulis excentricus* CB 48 uid55641 (NC_014816, NC_014817), *Maricaulis maris* MCS10 (NC_008347). The table indicates the absence of an homologous protein with the research criteria used (NA), the absence of a GANTC (0) or the presence of a GANTC (distance from the translational start site in bps; if more than one GANTC is located in the region, the motif closest to the translational start site is indicated). All sequences were downloaded from the NCBI FTP repository.

Order	Family	Species	Number of NGANTCN sites (sequences)
Caulobacterales	Caulobacteraceae	Brevundimonas_subvibrioides_ATCC_15264_uid42117	1 (CGACTCA)
Caulobacterales	Caulobacteraceae	Caulobacter_crescentus	2 (GGATTCT CGACTCA)
Caulobacterales	Caulobacteraceae	Caulobacter_crescentus_NA1000	2 (GGATTCT CGACTCA)
Caulobacterales	Caulobacteraceae	Caulobacter_K31	6 (TGAGTCG GGAGTCC GGAATCC TGATTCC CGACTCA TGACT[illegible]
Caulobacterales	Caulobacteraceae	Caulobacter_segnis_ATCC_21756_uid41709	1 (CGACTCA)
Caulobacterales	Caulobacteraceae	Phenylobacterium_zucineum_HLK1	0
Parvularculales	Parvularculaceae	Parvularcula_bermudensis_HTCC2503_uid51641	3 (TGACTCT CGATTCT GGAATCA)
Rhizobiales	Bartonellaceae	Bartonella_bacilliformis_KC583	0
Rhizobiales	Bartonellaceae	Bartonella_grahamii_as4aup	0
Rhizobiales	Bartonellaceae	Bartonella_henselae_Houston-1	0
Rhizobiales	Bartonellaceae	Bartonella_quintana_Toulouse	0
Rhizobiales	Bartonellaceae	Bartonella_tribocorum_CIP_105476	0
Rhizobiales	Beijerinckiaceae	Beijerinckia_indica_ATCC_9039	0
Rhizobiales	Beijerinckiaceae	Methylocella_silvestris_BL2	0
Rhizobiales	Bradyrhizobiaceae	Bradyrhizobium_BTAi1	0
Rhizobiales	Bradyrhizobiaceae	Bradyrhizobium_japonicum	1 (TGACTCC)
Rhizobiales	Bradyrhizobiaceae	Bradyrhizobium_ORS278	0
Rhizobiales	Bradyrhizobiaceae	Nitrobacter_hamburgensis_X14	0
Rhizobiales	Bradyrhizobiaceae	Nitrobacter_winogradskyi_Nb-255	1 (AGATTCC)
Rhizobiales	Bradyrhizobiaceae	Oligotropha_carboxidovorans_OM5	0
Rhizobiales	Bradyrhizobiaceae	Rhodopseudomonas_palustris_BisA53	0
Rhizobiales	Bradyrhizobiaceae	Rhodopseudomonas_palustris_BisB18	1 (CGATTCA)
Rhizobiales	Bradyrhizobiaceae	Rhodopseudomonas_palustris_BisB5	1 (TGACTCT)
Rhizobiales	Bradyrhizobiaceae	Rhodopseudomonas_palustris_CGA009	1 (TGACTCT)
Rhizobiales	Bradyrhizobiaceae	Rhodopseudomonas_palustris_HaA2	1 (TGACTCT)
Rhizobiales	Bradyrhizobiaceae	Rhodopseudomonas_palustris_TIE_1	1 (TGACTCT)
Rhizobiales	Brucellaceae	Brucella_abortus_bv__1_9_941	0
Rhizobiales	Brucellaceae	Brucella_abortus_S19	0
Rhizobiales	Brucellaceae	Brucella_canis_ATCC_23365	0
Rhizobiales	Brucellaceae	Brucella_melitensis_ATCC_23457	0
Rhizobiales	Brucellaceae	Brucella_melitensis_biovar_Abortus	0
Rhizobiales	Brucellaceae	Brucella_melitensis_bv__1_16M_uid180	0
Rhizobiales	Brucellaceae	Brucella_microti_CCM_4915	0
Rhizobiales	Brucellaceae	Brucella_ovis	0
Rhizobiales	Brucellaceae	Brucella_suis_1330	0
Rhizobiales	Brucellaceae	Brucella_suis_ATCC_23445	0
Rhizobiales	Brucellaceae	Ochrobactrum_anthropi_ATCC_49188	0
Rhizobiales	Hyphomicrobiaceae	Hyphomicrobium_denitrificans_ATCC_51888_uid50325	0
Rhizobiales	Methylobacteriaceae	Methylobacterium_4_46	0
Rhizobiales	Methylobacteriaceae	Methylobacterium_chloromethanicum_CM4	0
Rhizobiales	Methylobacteriaceae	Methylobacterium_extorquens_AM1	0
Rhizobiales	Methylobacteriaceae	Methylobacterium_extorquens_DM4	0
Rhizobiales	Methylobacteriaceae	Methylobacterium_extorquens_PA1	0
Rhizobiales	Methylobacteriaceae	Methylobacterium_nodulans_ORS_2060	0
Rhizobiales	Methylobacteriaceae	Methylobacterium_populi_BJ001	0
Rhizobiales	Methylobacteriaceae	Methylobacterium_radiotolerans_JCM_2831	1 (GGAATCC)
Rhizobiales	Phyllobacteriaceae	Mesorhizobium_BNC1	0
Rhizobiales	Phyllobacteriaceae	Mesorhizobium_loti	0
Rhizobiales	Phyllobacteriaceae	Parvibaculum_lavamentivorans_DS-1	0
Rhizobiales	Rhizobiaceae	Agrobacterium_radiobacter_K84	0
Rhizobiales	Rhizobiaceae	Agrobacterium_tumefaciens_C58_Cereon	1 (AGAATCG)
Rhizobiales	Rhizobiaceae	Agrobacterium_tumefaciens_C58_Cereon	1 (TGATTCG)
Rhizobiales	Rhizobiaceae	Agrobacterium_vitis_S4	1 (CGATTCG)
Rhizobiales	Rhizobiaceae	Candidatus_Liberibacter_asiaticus_psy62	1 (TGACTCT)
Rhizobiales	Rhizobiaceae	Rhizobium_etli_CFN_42	0
Rhizobiales	Rhizobiaceae	Rhizobium_etli_CIAT_652	0
Rhizobiales	Rhizobiaceae	Rhizobium_leguminosarum_bv__trifolii_WSM1325	0
Rhizobiales	Rhizobiaceae	Rhizobium_leguminosarum_bv__trifolii_WSM2304	0
Rhizobiales	Rhizobiaceae	Rhizobium_leguminosarum_bv_viciae_3841	0
Rhizobiales	Rhizobiaceae	Rhizobium_NGR234	0
Rhizobiales	Rhizobiaceae	Sinorhizobium_medicae_WSM419	0
Rhizobiales	Rhizobiaceae	Sinorhizobium_meliloti	0
Rhizobiales	Xanthobacteraceae	Azorhizobium_caulinodans_ORS_571	1 (GGATTCG)
Rhizobiales	Xanthobacteraceae	Starkeya_novella_DSM_506_uid48815	0
Rhizobiales	Xanthobacteraceae	Xanthobacter_autotrophicus_Py2	1 (GGATTCG)
Rhodobacterales	Hyphomonadaceae	Hirschia_baltica_ATCC_49814	0
Rhodobacterales	Hyphomonadaceae	Hyphomonas_neptunium_ATCC_15444	0
Rhodobacterales	Hyphomonadaceae	Maricaulis_maris_MCS10	1 (TGAATCG)
Rhodobacterales	Rhodobacteraceae	Dinoroseobacter_shibae_DFL_12	2 (CGAATCG AGACTCA)
Rhodobacterales	Rhodobacteraceae	Jannaschia_CCS1	0
Rhodobacterales	Rhodobacteraceae	Paracoccus_denitrificans_PD1222	2 (CGAATCT GGATTCG)
Rhodobacterales	Rhodobacteraceae	Rhodobacter_capsulatus_SB_1003_uid47509	2 (CGATTCG GGACTCC)
Rhodobacterales	Rhodobacteraceae	Rhodobacter_sphaeroides_2_4_1	1 (CGACTCC)
Rhodobacterales	Rhodobacteraceae	Rhodobacter_sphaeroides_ATCC_17025	1 (CGACTCC)
Rhodobacterales	Rhodobacteraceae	Rhodobacter_sphaeroides_ATCC_17029	1 (CGACTCC)
Rhodobacterales	Rhodobacteraceae	Rhodobacter_sphaeroides_KD131	1 (CGACTCC)
Rhodobacterales	Rhodobacteraceae	Roseobacter_denitrificans_OCh_114	1 (CGAATCG)
Rhodobacterales	Rhodobacteraceae	Ruegeria_pomeroyi_DSS_3	1 (AGATTCT)
Rhodobacterales	Rhodobacteraceae	Silicibacter_TM1040	1 (AGACTCA)
Rhodospirillales	Acetobacteraceae	Acetobacter_pasteurianus_IFO_3283_01	0
Rhodospirillales	Acetobacteraceae	Acidiphilium_cryptum_JF-5	0
Rhodospirillales	Acetobacteraceae	Gluconacetobacter_diazotrophicus_PAl_5_FAPERJ	3 (TGATTCA CGAATCG CGACTCG)
Rhodospirillales	Acetobacteraceae	Gluconacetobacter_diazotrophicus_PAl_5_JGI	3 (TGATTCA CGAATCG CGACTCG)
Rhodospirillales	Acetobacteraceae	Gluconobacter_oxydans_621H	0
Rhodospirillales	Acetobacteraceae	Granulobacter_bethesdensis_CGDNIH1	0
Rhodospirillales	Rhodospirillaceae	Azospirillum_B510_uid46085	1 (CGATTCC)
Rhodospirillales	Rhodospirillaceae	Magnetospirillum_magneticum_AMB-1	1 (CGAATCG)
Rhodospirillales	Rhodospirillaceae	Rhodospirillum_rubrum_ATCC_11170	1 (TGAGTCC)
Sphingomonadales	Erythrobacteraceae	Erythrobacter_litoralis_HTCC2594	2 (CGATTCA TGAATCG)
Sphingomonadales	Sphingomonadaceae	Novosphingobium_aromaticivorans_DSM_12444	1 (TGATTCG)
Sphingomonadales	Sphingomonadaceae	Sphingobium_japonicum_UT26S_uid47077	0
Sphingomonadales	Sphingomonadaceae	Sphingomonas_wittichii_RW1	3 (TGATTCG CGAATCG AGATTCG)
Sphingomonadales	Sphingomonadaceae	Sphingopyxis_alaskensis_RB2256	1 (TGATTCC)
Sphingomonadales	Sphingomonadaceae	Zymomonas_mobilis_NCIMB_11163	0
Sphingomonadales	Sphingomonadaceae	Zymomonas_mobilis_ZM4	0

Table S2: Conservation of GANTC sites in the *ftsZ* promoter of *Alphaproteobacteria.* GANTC sites were searched in the intergenic region upstream of the sequence encoding the best protein blast hit for the *C. crescentus* NA1000 FtsZ protein in all complete Alphaproteobacterial proteomes available at the NCBI repository in January 2011. The table indicates the NCBI order and family for the species, the number of GANTC sites found in the upstream intergenic region and a 7 nucelotide sequence centered on the GANTC when a GANTC was found.

Presence of a GANTC site in the *mipZ* promoter in *Alphaproteobacteria*

Order	Family	Species	Number of NGANTCN sites (sequence)
Caulobacterales	Caulobacteraceae	Brevundimonas_subvibrioides_ATCC_15264_uid42117	2 (CGATTCT TGAGTCG)
Caulobacterales	Caulobacteraceae	Caulobacter_crescentus	2 (AGAGTCG TGAGTCG)
Caulobacterales	Caulobacteraceae	Caulobacter_crescentus_NA1000	2 (AGAGTCG TGAGTCG)
Caulobacterales	Caulobacteraceae	Caulobacter_K31	2 (AGAGTCG CGAGTCG)
Caulobacterales	Caulobacteraceae	Caulobacter_segnis_ATCC_21756_uid41709	1 (CGAGTCG)
Caulobacterales	Caulobacteraceae	Phenylobacterium_zucineum_HLK1	0
Parvularculales	Parvularculaceae	Parvularcula_bermudensis_HTCC2503_uid51641	0
Rhizobiales	Bradyrhizobiaceae	Bradyrhizobium_BTAi1	1 (TGACTCG)
Rhizobiales	Bradyrhizobiaceae	Bradyrhizobium_japonicum	2 (GGACTCA TGAATCG)
Rhizobiales	Bradyrhizobiaceae	Bradyrhizobium_ORS278	2 (GGATTCA TGACTCG)
Rhizobiales	Phyllobacteriaceae	Parvibaculum_lavamentivorans_DS-1	1 (CGATTCG)
Rhizobiales	Bradyrhizobiaceae	Rhodopseudomonas_palustris_BisA53	1 (GGATTCA)
Rhizobiales	Bradyrhizobiaceae	Rhodopseudomonas_palustris_BisB5	2 (GGACTCG CGATTCA)
Rhizobiales	Bradyrhizobiaceae	Rhodopseudomonas_palustris_CGA009	2 (GGAATCC GGATTCA)
Rhizobiales	Bradyrhizobiaceae	Rhodopseudomonas_palustris_HaA2	1 (GGATTCA)
Rhizobiales	Bradyrhizobiaceae	Rhodopseudomonas_palustris_TIE_1	2 (GGAATCC GGATTCA)
Rhodobacterales	Rhodobacteraceae	Dinoroseobacter_shibae_DFL_12	1 (GGATTCC)
Rhodobacterales	Hyphomonadaceae	Hirschia_baltica_ATCC_49814	0
Rhodobacterales	Hyphomonadaceae	Hyphomonas_neptunium_ATCC_15444	0
Rhodobacterales	Rhodobacteraceae	Jannaschia_CCS1	0
Rhodobacterales	Hyphomonadaceae	Maricaulis_maris_MCS10	0
Rhodobacterales	Rhodobacteraceae	Paracoccus_denitrificans_PD1222	3 (CGACTCA CGATTCG GGATTCG)
Rhodobacterales	Rhodobacteraceae	Rhodobacter_capsulatus_SB_1003_uid47509	1 (GGATTCG)
Rhodobacterales	Rhodobacteraceae	Rhodobacter_sphaeroides_2_4_1	0
Rhodobacterales	Rhodobacteraceae	Rhodobacter_sphaeroides_ATCC_17025	0
Rhodobacterales	Rhodobacteraceae	Rhodobacter_sphaeroides_ATCC_17029	0
Rhodobacterales	Rhodobacteraceae	Rhodobacter_sphaeroides_KD131	0
Rhodobacterales	Rhodobacteraceae	Roseobacter_denitrificans_OCh_114	1 (TGATTCA)
Rhodobacterales	Rhodobacteraceae	Ruegeria_pomeroyi_DSS_3	0
Rhodobacterales	Rhodobacteraceae	Silicibacter_TM1040	1 (TGATTCG)
Rhodospirillales	Rhodospirillaceae	Magnetospirillum_magneticum_AMB-1	0
Rhodospirillales	Rhodospirillaceae	Rhodospirillum_rubrum_ATCC_11170	1 (AGATTCA)
Sphingomonadales	Erythrobacteraceae	Erythrobacter_litoralis_HTCC2594	0
Sphingomonadales	Sphingomonadaceae	Novosphingobium_aromaticivorans_DSM_12444	0
Sphingomonadales	Sphingomonadaceae	Sphingobium_japonicum_UT26S_uid47077	0
Sphingomonadales	Sphingomonadaceae	Sphingomonas_wittichii_RW1	0
Sphingomonadales	Sphingomonadaceae	Sphingopyxis_alaskensis_RB2256	0
Sphingomonadales	Sphingomonadaceae	Zymomonas_mobilis_NCIMB_11163	0
Sphingomonadales	Sphingomonadaceae	Zymomonas_mobilis_ZM4	0

Table S3: Conservation of GANTC sites in the *mipZ* promoter of *Alphaproteobacteria*. GANTC sites were searched in the intergenic region upstream of the sequence encoding the best protein blast hit for the *C. crescentus* NA1000 MipZ protein in all complete Alphaproteobacterial proteomes available at the NCBI repository in January 2011. The table indicates the NCBI order and family for the species, the number of GANTC found in the uspream intergenic region and a 7 nucleotide sequence centered on the GANTC when a GANTC was found.

2. Supplementary Material and Methods

Table S4 : Oligonucleotides	
P*ftsZ*For	GGA ATT CGC CTG GCT GCG CGA CAA TCT CTA A
P*ftsZ*Rev	AAC TGC AGG CCG CGC TCA TGC CCA CCT C
P*ftsZ*-CG-For	GCT CGA CCG GAC CAC GAC TGA GAT TAT GCA GTT AAC C
P*ftsZ*-CG-Rev	GGT TAA CTG CAT AAT CTC AGT CGT GGT CCG GTC GAG C
P*ftsZ*-NG-For	GCT CGA CCG GAC CAC GAG TCA GAT TAT GCA GTT AAC C
P*ftsZ*-NG-Rev	GGT TAA CTG CAT AAT CTG ACT CGT GGT CCG GTC GAG C
P*ftsZ*-AT-For	GCT CGA CCG GAC CAC GTC TCA GAT TAT GCA GTT AAC C
P*ftsZ*-AT-Rev	GGT TAA CTG CAT AAT CTG AGA CGT GGT CCG GTC GAG C
ftsZ_Pr_SalIF_F	GCT CGA CCG GAC GTC GAC TCA GAT TAT GCA GTT AAC C
ftsZ_Pr_SalIF_R	GGT TAA CTG CAT AAT CTG AGT CGA CGT CCG GTC GAG C
mipZP_GC_rev	AAC TGC AGG GCT CGG ATC CTT CTG CGT CGC GAC TGA GCG ACT CTT AAT CGA AGG
mipZP_NC_rev	AAC TGC AGG GCT CGG ATC CTT CTG CGT CGC GAG TCA GCG ACT CTT AAT CGA AGG
mipZP_AT_rev	AAC TGC AGG GCT CGG ATC CTT CTG CGT CGC GAC ACA GCG ACT CTT AAT CGA AGG
PmipZ-SalIR-Rev	AAC TGC AGG GCT CGG ATC CTT CTG CGT GTC GAC TCA GCG ACT CTT AAT CGA AGG
PftsZ_DC_F	CGG TCC CGC TCG ACC GGA CCA CGA CTC AGA TTA TGC AGA ATT CCG CCG ATT AAC GAT GG
PftsZ_DC_R	GTG GTC CGG TCG AGC GGG ACC GTA CGT TAA ATT GTG GTC TAA CAA GTG AAT CCC CCA ATG AAC CAA AGG
salIM_CS_F	GGA ATT CCA TAT GCA TAG CGA GGC GAG AGA G
salIM_CS_R	CGA CGC GTT CAC CAC AAA GCG ACT TGA TCC
OmegaFR	CGA CGC GTG AAT TCC CGG GGA TCC GGT GAT TGA TTG AGC
qPCR-*ftsZ*-For	ATC GCT AAC CCG CTG CTG GAC
qPCR-*ftsZ*-Rev	CGC GCC GAA GAT GAT GTT GG
qPCR-*mipZ*-For	GCG CGT GGC TGG ACA ACA AGA
qPCR-*mipZ*-Rev	TTC GAA ACC GGC CAC CTG CTC
qPCR-*CC3527*-For	TCA CGC CCG CGA GGA TTT C
qPCR-*CC3527*-Rev	CGT TGC TTC GGC GGG ATG TA

**Table S5: Bacterial strains**		
**Name**	**Genotype**	**Construction or reference**
Escherichia coli		
EC100	*F- mcrA Δ(mrr-hsdRMS-mcrBC) Φ80dlacZΔM15 ΔlacX74 recA1 endA1 araD139 Δ(ara, leu)7697 galU galK λ- rpsL (StrR) nupG*	Epicentre.
Caulobacter crescentus		
NA1000	Synchronizable derivative of the wild-type strain CB15	(Evinger & Agabian, 1977)
LS2144	NA1000 Δ*ccrM*::Ω pCS226	(Stephens *et al.*, 1996)
YB1585	NA1000 *ftsZ*::P*xyl*X::*ftsZ*	(Wang *et al.*, 2001)
JOE2321	NA1000 Δ*lacA*	(Arellano *et al.*, 2010)
GM1258	NA1000 *trpE*::*Tn5*Ω-MP	(Marczynski, 1999)
LS2293	NA1000 *hrcA*Ω	(Roberts *et al.*, 1996)
MT114	NA1000 *xylX*::P*xyl*X::*ftsZ-YFP*	M. Thanbichler, unpublished.
JC1149	NA1000 Δ*ccrM*::Ω	This work.
JC948	NA1000 *ftsZ*::P*xyl*X::*ftsZ* Δ*ccrM*::Ω	This work.
JC1206	NA1000 *xylX*::P*xyl*X::*ftsZ-YFP* Δ*ccrM*::Ω	This work.
JC1084	NA1000 *xylX*::pXT-*M.salI*	This work.
JC835	NA1000 *xylX*::pXT	This work.
JC1147	NA1000 *xylX*::pXT-*M.salI* Δ*ccrM*::Ω	This work.
JC1127	NA1000 *xylX*::pXT Δ*ccrM*::Ω	This work.
JC1222 to JC1233	NA1000 Δ*ccrM*::Ω strains with unidentified spontaneous suppressor mutations increasing the fitness of the strain in PYE.	This work.
JC1161	NA1000 P*ftsZ-salIF*	This work.
JC1169	NA1000 *xylX*::pXT-*M.salI* P*ftsZ-salIF*::*ftsZ* Δ*ccrM*::Ω	This work.
JC1168	NA1000 *xylX*::pXT P*ftsZ-salIF*::*ftsZ* Δ*ccrM*::Ω	This work.
JC1265	NA1000 Δ*lacA trpE*::*Tn5*Ω-MP	This work.
JC1266	NA1000 Δ*lacA hrcA*Ω	This work.
JC1269	NA1000 Δ*lacA trpE*::*Tn5*Ω-	This work.

	MP::pNPTS138-P*ftsZ*-WT-*lacZ*-Ω	
JC1270	NA1000 Δ*lacA trpE*::*Tn5*Ω-MP::pNPTS138-P*ftsZ*-C5-*lacZ*-Ω	This work.
JC1271	NA1000 Δ*lacA hrcA*Ω::pNPTS138-P*ftsZ*-WT-*lacZ*-Ω	This work.
JC1272	NA1000 Δ*lacA hrcA*Ω::pNPTS138-P*ftsZ*-C5-*lacZ*-Ω	This work.

**Table S6 : Plasmids**		
**Name**	**Description**	**Reference**
p*lacZ*290	Low copy number vector, usually used to create *lacZ* transcriptional fusion.	(Gober & Shapiro, 1992)
pSC226	*ccrM* gene under the control of the *xylX* promoter into p*lacZ*290.	(Stephens et al., 1996)
pNPTS138	Integrating plasmid containing the *sacB* gene.	D. Alley, unpublished.
pNPT228-*gcrA*P-*lacZ*-Ω	Plasmid used to amplify the Ω cassette.	(Collier *et al.*, 2006)
p*lacZ*290-P*ftsZ*-WT	Wild-type *ftsZ* promoter region cloned into p*lacZ290*.	This work
p*lacZ*290-P*ftsZ*-C5	Mutant *ftsZ* promoter region cloned into p*lacZ290*. GACTC mutated into GACTG.	This work.
p*lacZ*290-P*ftsZ*-N3	Mutant *ftsZ* promoter region cloned into p*lacZ290*. GACTC mutated into GAGTC.	This work.
p*lacZ*290-P*ftsZ*-A2	Mutant *ftsZ* promoter region cloned into p*lacZ290*. GACTC mutated into GTCTC.	This work.
p*lacZ*290-P*mipZ* (WT) (=p*lacZ*290-*mipZP)*	Wild-type *mipZ* promoter region cloned into p*lacZ290*.	(Fernandez-Fernandez et al., 2011)
p*lacZ*290-P*mipZ*-C5	Mutant *mipZ* promoter region cloned into p*lacZ290*. GAGTC mutated into GAGTG.	This work.
p*lacZ*290-P*mipZ*-N3	Mutant *mipZ* promoter region cloned into p*lacZ290*. GAGTC mutated into GACTC.	This work.
p*lacZ*290-P*mipZ*-A2	Mutant *mipZ* promoter region cloned into p*lacZ290*. GAGTC mutated into GTGTC.	This work.
p*lacZ*290-P*ftsZ-salIF*	Mutant *ftsZ* promoter region cloned into p*lacZ290*. CACGACTC mutated into GTCGACTC.	This work.
p*lacZ*290-P*mipZ-salIR*	Mutant *mipZ* promoter region cloned into p*lacZ290*. GAGTCGCG mutated into GAGTCGAC.	This work.

p*lacZ*290-P*ftsZ*-DC	Mutant *ftsZ* promoter region cloned into p*lacZ290*. Mutations disrupt a CtrA and two putative DnaA binding sites.	This work.
pXTCYC-4 = pXT	Expression plasmid containing a long *xylX* promoter for integration at the *xylX* promoter locus.	(Thanbichler *et al.*, 2007)
pXT-*M.salI*	*M.salI* sequence cloned into pXTCYC-4	This work.
pIJ4467	Plasmid encoding the entire SalI restriction-modification system.	(Alvarez *et al.*, 1993)
pNTPS138-P*ftsZ-salIF*	Plasmid used to mutate the *ftsZ* promoter at the native *ftsZ* locus by double recombination.	This work.
pNPTS138-P*ftsZ*-WT-*lacZ*-Ω	Plasmid containing the P*ftsZ*-WT-*lacZ* fusion from p*lacZ*290-P*ftsZ*-WT and the Ω cassette from pNPT228-*gcrA*P-*lacZ*-Ω.	This work.
pNPTS138-P*ftsZ*-C5-*lacZ*-Ω	Plasmid containing the P*ftsZ*-C5-*lacZ* fusion from p*lacZ*290-P*ftsZ*-C5 and the Ω cassette from pNPT228-*gcrA*P-*lacZ*-Ω.	This work.

Construction of plasmids and strains

Construction of the placZ*290-P*ftsZ *plasmids*. The *ftsZ* promoter was amplified from NA1000 genomic DNA using primers PftsZFor and PftsZRev, digested with EcoRI and PstI and cloned into p*lacZ*290 at the EcoRI and PstI sites. The *ftsZ* promoter variants with the point mutations in the GANTC motif (C5, N3 and A2 variants) were generated using a two-step PCR amplification procedure using NA1000 genomic DNA. Primers PftsZFor and PftsZRev were used for each construction, together with primers P*ftsZ*-CG-For and P*ftsZ*-CG-Rev for the C5 construct, P*ftsZ*-NG-For and P*ftsZ*-NG-Rev for the N3 construct, and P*ftsZ*-AT-For and P*ftsZ*-AT-Rev for the A2 construct. The *ftsZ* promoter variant with the *salIF* mutation was constructed with a similar procedure using primers PftsZFor, PftsZRev, ftsZ_Pr_SalIF_F and ftsZ_Pr_SalIF_R. The *ftsZ* promoter variant with the mutations at the CtrA and DnaA binding sites were constructed with a similar procedure using primers PftsZFor, PftsZRev, ftsZ_Pr_DC_F and ftsZ_Pr_DC_R.

Construction of the placZ*290-P*mipZ *plasmids.* The *mipZ* promoter variants with the point mutations in the GANTC motif (C5, N3 and A2 variants) or with the *salIR* mutation were PCR amplified using p*lacZ*290-P*mipZ*(WT) as a template. The forward primer previously used to construct p*lacZ*290-P*mipZ*(WT) (Fernandez-Fernandez *et al.*, 2011) was used for each construction, together with mipZP_GC_rev for the C5 construct, mipZP_NC_rev for the N3 construct, mipZP_AT_rev for the A2 construct and PmipZ-SalIR-Rev for the *salIR* construct. PCR products were digested with EcoRI and PstI and cloned into p*lacZ*290 at the EcoRI and PstI sites.

*Construction of pXT-*M.salI*.* The *M.salI* sequence was PCR-amplified from pIJ4467 plasmid using primers salIM_CS_F and salIM_CS_R. PCR products were digested with NdeI and MluI and cloned into NdeI-MluI-digested pXTCYC-4.

*Construction of pNTPS138-P*ftsZ-salIF*.* The *ftsZ-salIF* promoter region was PCR-amplified from p*lacZ*290-P*ftsZ-salIF* with primers PftsZFor and PftsZRev and cloned into pNTPS138 between the PstI and EcoRI sites.

Construction of pNPTS138-P*ftsZ*-WT-*lacZ*-Ω and pNPTS138-P*ftsZ*-C5-*lacZ*-Ω*.* The EcoRI-DraI fragments from p*lacZ*290-P*ftsZ*-WT or p*lacZ*290-P*ftsZ*-C5 were cloned into the EcoRI-EcoRV sites of pNPTS138 ; an Ω cassette was amplified from pNPT228-*gcrA*P-*lacZ*-Ω with the OmegaFR primer and cloned into the EcoRI site of the resulting plasmids.

Strains constructions.
Plasmids were introduced into *C. crescentus* strains by transformation. Electroporations of *p*lacZ*290 derivatives* were carried out at 2.5 kV, 100 ohms, 25 µF into *E. coli* EC100 and at 1.5 kV, 400 ohms, 25 µF for *C. crescentus* strains. Bacteriophage ΦCR30 was used for general transduction into *C. crescentus* (Ely, 1991).
To construct strains carrying the Δ*ccrM* mutation, the transduction of the Δ*ccrM* mutation (in minimal medium) was systematically the last step of the strain construction to reduce the risk of appearance of suppressor mutations.
To construct strain JC1161 (NA1000 P*ftsZ-salIF*), the pNTPS138-P*ftsZ-salIF* plasmid was introduced into NA1000 by electroporation and selection on kanamycin-containing plates; the resulting strain with pNTPS138-P*ftsZ-salIF* integrated at the *ftsZ* promoter locus was grown to stationary phase in PYE medium lacking kanamycin. Cells were plated on PYE + sucrose 3% and incubated at 28°C. Single colonies were picked and transferred in parallel onto plain PYE plates and PYE plates containing kanamycin. Kanamycin-sensitive clones, which had lost the integrated plasmid due to a single recombination event, were then tested for the presence of the mutated *ftsZ-salI* promoter upstream of the *ftsZ* coding sequence. pXT and pXT-*M.salI* were introduced into JC1161, before the Δ*ccrM* mutation was transduced into these strains.
To construct strains JC1265 and JC1266, the chromosomal loci containing the Ω cassettes from strains GM1258 and LS2293, respectively, were transduced into strain JOE2321, selecting for spectinomycin and spectromycin resistance. Plasmids pNPTS138-P*ftsZ*-WT-*lacZ*-Ω and pNPTS138-P*ftsZ*-C5-*lacZ*-Ω were introduced into JC1265 and JC1266 by electroporation, giving strains JC1269, JC1270, JC1271 and JC1272.

References:

Alvarez, M.A., K.F. Chater & M.R. Rodicio, (1993) Complex transcription of an operon encoding the SalI restriction-modification system of *Streptomyces albus* G. *Mol Microbiol* **8**: 243-252.

Arellano, B.H., J.D. Ortiz, J. Manzano & J.C. Chen, (2010) Identification of a dehydrogenase required for lactose metabolism in *Caulobacter crescentus*. *Appl Environ Microbiol* **76**: 3004-3014.

Collier, J., S.R. Murray & L. Shapiro, (2006) DnaA couples DNA replication and the expression of two cell cycle master regulators. *Embo J* **25**: 346-356.

Ely, B., (1991) Genetics of *Caulobacter crescentus*. *Methods Enzymol* **204**: 372-384.

Evinger, M. & N. Agabian, (1977) Envelope-associated nucleoid from *Caulobacter crescentus* stalked and swarmer cells. *J Bacteriol* **132**: 294-301.

Fernandez-Fernandez, C., D. Gonzalez & J. Collier, (2011) Regulation of the Activity of the Dual-Function DnaA Protein in *Caulobacter crescentus*. *PLoS One* **6**: e26028.

Gober, J.W. & L. Shapiro, (1992) A developmentally regulated *Caulobacter* flagellar promoter is activated by 3' enhancer and IHF binding elements. *Mol Biol Cell* **3**: 913-926.
Goley, E.D., Y.C. Yeh, S.H. Hong, M.J. Fero, E. Abeliuk, H.H. McAdams & L. Shapiro, (2011) Assembly of the *Caulobacter* cell division machine. *Mol Microbiol* **80**: 1680-1698.
Marczynski, G.T., (1999) Chromosome methylation and measurement of faithful, once and only once per cell cycle chromosome replication in *Caulobacter crescentus*. *J Bacteriol* **181**: 1984-1993.
Roberts, R.C., C.D. Mohr & L. Shapiro, (1996) Developmental programs in bacteria. *Curr Top Dev Biol* **34**: 207-257.
Stephens, C., A. Reisenauer, R. Wright & L. Shapiro, (1996) A cell cycle-regulated bacterial DNA methyltransferase is essential for viability. *Proc Natl Acad Sci U S A* **93**: 1210-1214.
Thanbichler, M., A.A. Iniesta & L. Shapiro, (2007) A comprehensive set of plasmids for vanillate- and xylose-inducible gene expression in *Caulobacter crescentus*. *Nucleic Acids Res* **35**: e137.
Wang, Y., B.D. Jones & Y.V. Brun, (2001) A set of *ftsZ* mutants blocked at different stages of cell division in *Caulobacter*. *Mol Microbiol* **40**: 347-360.
Zweiger, G., G. Marczynski & L. Shapiro, (1994) A *Caulobacter* DNA methyltransferase that functions only in the predivisional cell. *J Mol Biol* **235**: 472-485.
